# Systematic Review of the Toxicity of Long-Course Oral Corticosteroids in Children

**DOI:** 10.1371/journal.pone.0170259

**Published:** 2017-01-26

**Authors:** Fahad Aljebab, Imti Choonara, Sharon Conroy

**Affiliations:** Division of Medical Sciences & Graduate Entry Medicine, School of Medicine, University of Nottingham, Royal Derby Hospital Centre, Derby, United Kingdom; York University, UNITED KINGDOM

## Abstract

**Background:**

Long courses of oral corticosteroids are commonly used in children in the management of chronic conditions. Various adverse drug reactions (ADRs) are known to occur with their use. This systematic review aimed to identify the most common and serious ADRs and to determine their relative risk levels.

**Methods:**

A literature search of Embase, Medline, International Pharmaceutical Abstracts, CINAHL, Cochrane Library and PubMed was performed with no language restrictions in order to identify studies where oral corticosteroids were administered to patients aged 28 days to 18 years of age for at least 15 days of treatment. Each database was searched from their earliest dates to January 2016. All studies providing clear information on ADRs were included.

**Results:**

One hundred and one studies including 33 prospective cohort studies; 21 randomised controlled trials; 21 case series and 26 case reports met the inclusion criteria. These involved 6817 children and reported 4321 ADRs. The three ADRs experienced by the highest number of patients were weight gain, growth retardation and Cushingoid features with respective incidence rates of 21.1%, 18.1% and 19.4% of patients assessed for these ADRs. 21.5% of patients measured showed decreased bone density and 0.8% of patients showed osteoporosis. Biochemical HPA axis suppression was detected in 269 of 487 patients where it was measured. Infection was the most serious ADR, with twenty one deaths. Varicella zoster was the most frequent infection (9 deaths).

**Conclusions:**

Weight gain, growth retardation and Cushingoid features were the most frequent ADRs seen when long-course oral corticosteroids were given to children. Increased susceptibility to infection was the most serious ADR.

## Introduction

Corticosteroids are used widely for their immunosuppressant and anti-inflammatory properties. They may be used individually or in combination with other drugs and are prescribed in both short and long courses depending on the condition being treated and the response of the patient [1]. The adverse effects from short-course use have been described recently and include changes in mood and behaviour, vomiting and sleep disturbance [2]. Long-course use of corticosteroids may lead to additional side effects [3]. Many of the side effects are reversible if the medication is stopped, while others may be permanent [4][5].

It is known that higher doses of corticosteroids, particularly when used for prolonged courses can cause hypothalamic-pituitary-adrenal axis (HPA axis) suppression. This can result in increased susceptibility to infections including viral infections such as varicella zoster, bacterial infections such as cellulitis and fungal infections such as candida, tinea, etc. [6].

The risk of specific adverse drug reactions (ADRs) following long term corticosteroids in children is unknown and these effects are of major concern. We therefore performed a systematic review to determine the toxicity associated with long course oral corticosteroids, to identify the most common and serious ADRs and to determine their relative risk levels. We defined long-course oral corticosteroids as use for at least 15 days.

## Methods

A systematic literature search was performed to identify all papers describing toxicity of corticosteroids in children. Six databases were searched up to January 2016: MEDLINE, EMBASE, International Pharmaceutical Abstracts, CINAHL, Cochrane Library and PubMed. The databases were searched separately and combined together to remove duplications. The search strategy included all languages and involved the keywords "prednis* or dexamethasone or betamethasone" which are the most frequent medications given orally to children for long periods. "Corticosteroid" was added to cover all other oral corticosteroid medication. The keywords "toxicity* or adverse drug reaction* or adverse event* or side effect* or adverse effect*" were used as recommended by the BMC Medical Research Methodology for systematic reviews of adverse effects [7]. Additionally, the keyword ‘‘safety*”, was used as in a previous systematic review [8]. The terms recommended by search strategies for Medline were used to cover the paediatric age group ‘‘child* or children* or p*ediatric* or infant* or adolescent*” [9]. The terms neonate, newborn and gestation were excluded. The keywords: oral, tablet, syrup and PO (abbreviation meaning by mouth) were used to cover all possibilities of oral administration of corticosteroids.

Inclusion criteria were original research studies assessing corticosteroid toxicity in children from 28 days up to 18 years of age. These included randomised controlled trials (RCTs), case series, case reports, cohort studies and letters. Five percent of randomly selected abstracts were independently assessed for eligibility by a second reviewer for assurance that no relevant studies were missed. Data including the number of patients, drug name, duration, diagnosis, number and type of ADRs were extracted.

Exclusion criteria included; review articles, editorials, studies that did not give ADR data, studies in adults, studies involving adults and children where paediatric data was not presented separately and studies in which corticosteroids were not given orally and/or were administered for less than 15 days.

The quality of included RCTs was assessed using the Cochrane collaborations tool for assessing risk of bias in randomised trials [10]. Any study showing a high risk of bias on three or more parameters was thereafter excluded. Prospective cohort studies were assessed using the Strengthening the Reporting of Observational Studies in Epidemiology (STROBE) checklist [11], where a score of over 70% is required for inclusion. Other studies were quality assessed using the Health Technology Assessment checklist [12]. All studies achieving at least satisfactory criteria were included. All quality assessments were independently assessed for eligibility and scored by two reviewers using a specially designed form. Any discrepancies were resolved by a third reviewer.

The incidence of ADRs was calculated from the RCTs and prospective cohort studies when more than one study reported the ADR.

Meta-analysis of included RCTs was performed using RevMan 5.3, software provided by Cochrane, used for organising the quality assessment for RCTs and for carrying out meta-analysis. Chi^2^ analysis of the difference between treatment duration periods (short- and long-course) was performed for the discussion section. P-values <0.05 were considered statistically significant.

## Results

**Fig 1:**
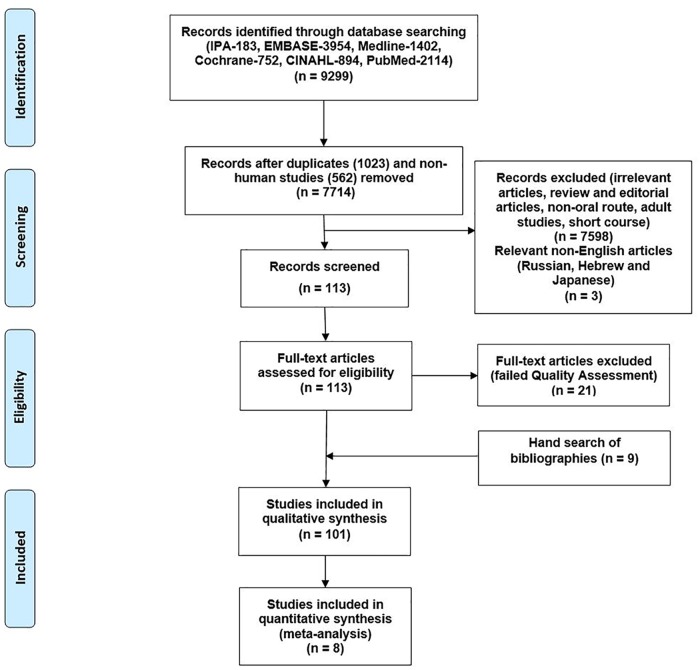
Flow chart of the literature search performed (PRISMA flow diagram).

7,714 articles were identified in total. 101 articles met the inclusion criteria after nine papers were added from manual searches of bibliographies, [13–33] [34–66] [67–87] [88–113] (S1 Appendix 1). The remaining articles were excluded for the reasons shown in Fig 1. Twenty one articles were excluded after quality assessment. Only one RCT contained four criteria with high risks of bias, and this study was excluded from the results [114] (Schaefer 2008, Fig 2). Twelve studies scoring < 70% in the STROBE checklist (range 30–57.5%) were excluded [115–126]. Eight case series were excluded for poor ratings in the Health Technology Assessment checklist [127–134].

**Fig 2:**
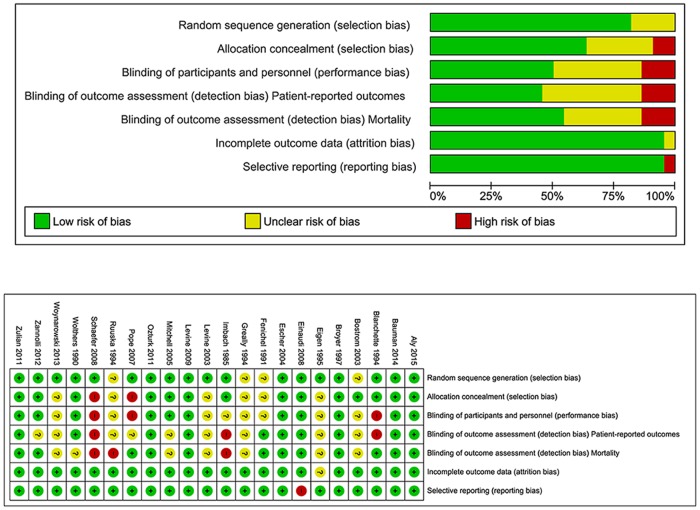
Risk of bias summary and graph.

Corticosteroids were used to manage a wide variety of medical conditions, of which the most common were leukaemia, haemangioma, asthma, nephrotic syndrome, cystic fibrosis and Crohn’s disease. Prednisolone and dexamethasone were the most commonly used drugs.

The studies included a total of 6817 patients and 4321 ADRs (Table 1). RCTs accounted for over half of the studies and patients (n = 3484) and one third (34.8%) of ADRs [13–33]. The 33 prospective cohort studies included 43% of patients (n = 2936) but reported half of the ADRs [34–66]. Prospective cohort studies detected more ADRs per patient than RCTs.

**Table 1:** Summary of included articles.

Type of Study	No. of Studies	No. of Patients						Total	No. of ADRs						Total
		Pred	Dexa	Bud	Methyl	Def	Beta		Pred	Dexa	Bud	Methyl	Def	Beta	
RCT*	**21**	1947	1352	130	22	20	13	**3484**	946	220	226	32	52	21	**1497**
**Prospective Cohort Studies (PCS)**	**33**	2834	102	-	-	-	-	**2936**	1795	355	-	-	-	-	**2150**
**Case Series (CS)**	**21**	255	66	23	21	-	-	**365**	335	203	26	42	-	-	**606**
**Case Report (CR)**	**26**	28	3	1	-	-	-	**32**	60	6	2	-	-	-	**68**
**Total**	**101**	**5064**	**1523**	**154**	**43**	**20**	**13**	**6817**	**3136**	**784**	**254**	**74**	**52**	**21**	**4321**¶

Abbreviation: Pred, Prednisolone; Dex, Dexamethasone; Bud, Budesonide; Methyl, Methylprednisolone; Def, Deflazacort; Beta, Betamethasone.

* Only data from patients taking oral corticosteroids were included.

^¶^Some patients had more than one adverse drug reaction

- Forty two studies looked for all possible symptomatic ADRs [13–28,30–41,44,47,48,52,53,55–62,64]. Fourteen of them also evaluated HPA axis suppression specifically [14–16,22,32,36,41,51–53,55–58]. Twenty five measured the weight [13,14,16–18,20–27,30–34,38,39,41,57–59,62], nineteen measured growth [19–22,27,30,32–35,37–41,57,59,61,62], eighteen evaluated blood pressure [20–22,24,27,31–35,37–39,44,47,59,60,62], fourteen evaluated blood glucose [20–22,26–28,30–33,39,59,60,62], and one measured bone mineral density [21].
- Three studies evaluated HPA axis suppression only [29][54][66].
- Two studies measured growth only [43][45].
- Two studies evaluated behavioural change and sleep disturbance only [50][65].
- One study evaluated both blood pressure and osteoporosis [49].
- One study measured weight only [46].
- One study measured bone mineral density only [42].
- One study evaluated infection only [63].

Thirty six different ADRs were reported from the RCTs and prospective cohort studies (Tables 2 and 3). In terms of numbers of patients experiencing them the three most common ADRs were weight gain, growth retardation and Cushingoid features, affecting between 21.1 to 18.1% of children.

**Table 2:** Observed/reported ADRs from RCTs and prospective cohort studies.

Adverse effects	No. of pt with ADRs¶	No. of studies reporting ADR	Denominators		Incidence (%)
			No. of studies looking for ADR	Total no. of patients	
**Cushingoid features**	**353**	**16**	**20**	**1817**	**19.4**
**Increased appetite**	**42**	**3**	**8**	**227**	**18.5**
**Gastrointestinal upset**	**86**	**6**	**16**	**543**	**15.8**
**Hirsutism**	**42**	**5**	**9**	**347**	**12.1**
**Acne**	**34**	**6**	**8**	**314**	**10.8**
**Gastric wall abnormality**	**163**	**5**	**16**	**1573**	**10.4**
**Insomnia**	**153**	**9**	**16**	**1538**	**9.9**
**Skin atrophy**	**123**	**2**	**9**	**1356**	**9.1**
**Infection**	**278**	**22**	**33**	**3203**	**8.7**
**Behavioural changes**	**257**	**14**	**19**	**3164**	**8.1**
**Rash**	**20**	**2**	**10**	**342**	**5.8**
**Skin striae**	**12**	**4**	**8**	**270**	**4.4**
**Headache**	**15**	**3**	**8**	**349**	**4.3**
**Fever**	**9**	**2**	**8**	**285**	**3.2**
**Myopathy**	**89**	**6**	**10**	**2974**	**3.0**
**Hair loss**	**6**	**2**	**6**	**217**	**2.8**
**Bruising easily**	**5**	**2**	**5**	**226**	**2.2**
**Fatigue**	**58**	**4**	**10**	**2974**	**2.0**
**Peripheral oedema**	**2**	**2**	**8**	**254**	**0.8**
**Hypopigmentation**	**108**	**1**	**-**	**-**	**-**
**Anorexia**	**3**	**1**	**-**	**-**	**-**
**Ulceration (arterial bleed)**	**1**	**1**	**-**	**-**	**-**
**Unknown**	**11**	**2**	**-**	**-**	**-**

^¶^Some patients had more than one adverse drug reaction

**Table 3:** ADRs from RCTs and prospective cohort studies requiring specific measurements.

Adverse effects	No. of pt with ADRs¶	No. of studies reporting ADR	Denominators		Incidence (%)
			No. of studies looking for ADR	Total no. of patients	
**HPA axis suppression**	**267**	**17**	**17**	**456**	**58.6**
**Decreased bone density**	**197**	**2**	**2**	**917**	**21.5**
**Weight gain**	**556**	**21**	**26**	**2636**	**21.1**
**Growth retardation**	**358**	**13**	**21**	**1983**	**18.1**
**Cataracts**	**25**	**5**	**8**	**424**	**5.9**
**Hypertension**	**176**	**14**	**19**	**3164**	**5.6**
**Hyperglycaemia**	**112**	**7**	**14**	**3272**	**3.4**
**Weight loss**	**33**	**3**	**26**	**2636**	**1.3**
**Osteoporosis**	**21**	**5**	**5**	**2552**	**0.8**
**Avascular necrosis**	**18**	**2**	**2**	**2663**	**0.7**
**Pancreatitis**	**9**	**1**	**-**	**-**	**-**
**Hypernatraemia**	**3**	**1**	**-**	**-**	**-**
**Hypokalaemia**	**1**	**1**	**-**	**-**	**-**
**Hypercholesterolaemia**	**1**	**1**	**-**	**-**	**-**

^¶^Some patients had more than one adverse drug reaction

**Fig 3:**
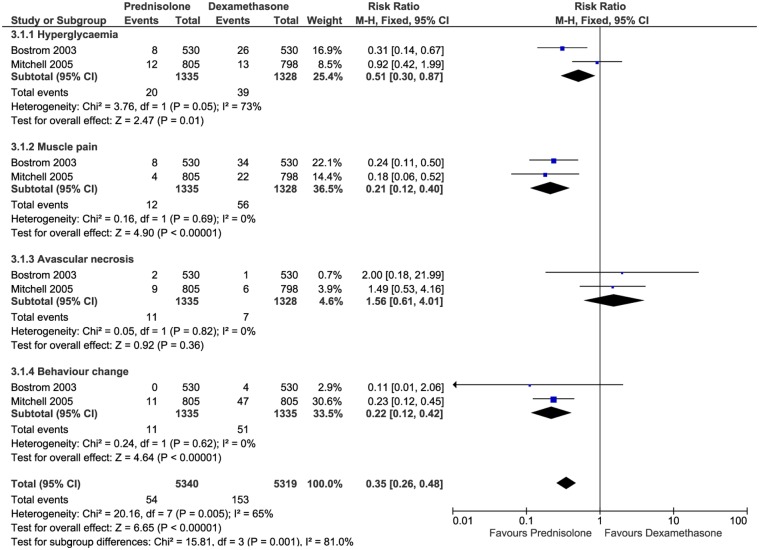
Relative risk of ADRs between prednisolone and dexamethasone for leukaemia patients.

**Fig 4:**
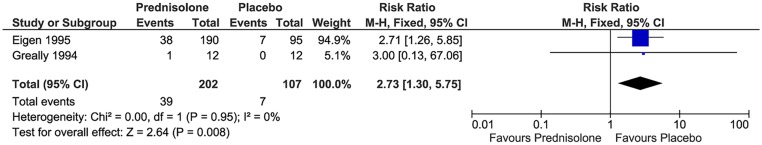
Relative risk of hyperglycaemia between prednisolone and placebo in cystic fibrosis patients.

Six RCTs and one prospective cohort study reported that 112 children experienced hyperglycaemia during treatment periods (incidence 3.4%) [20,22,27,28,30,31,60]. Two cases series and two case reports also reported 22 children who experienced hyperglycaemia [67][76][90][92]. The risk of hyperglycaemia was more frequent among older children (>10 years) [27][28]. Some patients with leukaemia and nephrotic syndrome required daily insulin [27][28][90]. Meta-analysis across two RCTs [27][28] showed statistically significant hyperglycaemia associated with oral dexamethasone compared to oral prednisolone in leukaemic patients (Fixed model, I^2^ = 73%, P = 0.01, RR = 0.51). (Fig 3). Meta-analysis across two RCTs [30][31] showed statistically significant hyperglycaemia associated with oral prednisolone compared to placebo in cystic fibrosis patients (Fixed model, I^2^ = 0%, P = 0.008, RR = 2.73). (Fig 4).

**Fig 5:**
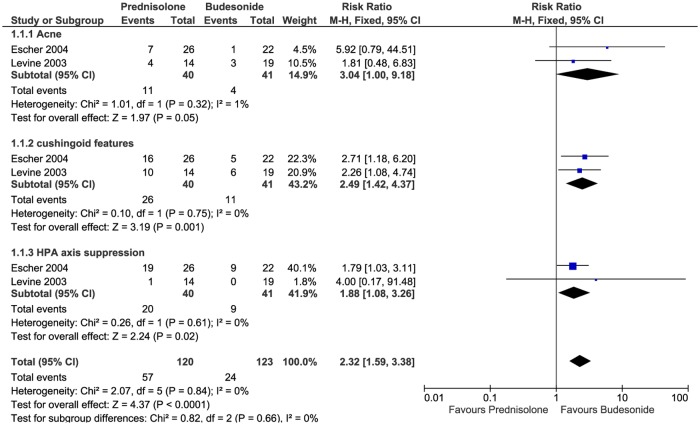
Relative risk of ADRs between prednisolone and budesonide in crohn's disease patients.

Seventeen studies (eleven prospective cohort studies and six RCTs) and two case series specifically evaluated the HPA axis [14–16,22,29,32,36,41,51–58,66,81,84] 269 of the 487 patients tested showed biochemical HPA axis suppression. Meta-analysis across two RCTs [14][15] showed statistically significant biochemical HPA axis suppression associated with oral prednisolone compared to oral budesonide (Fixed model, I^2^ = 0%, P = 0.02, RR = 1.88). (Fig 5). Some patients did not achieve adequate adrenal function (cortisol level >18 μg/dL) until eight weeks after completion of therapy with dexamethasone [57]. Also some children still had adrenal suppression (cortisol level <18 μg/dL) after withdrawal of dexamethasone for up to 4–8 months [58]. One infant with a haemangioma withdrew from treatment with oral prednisolone due to adrenal crisis [36].

Twenty six RCTs and cohort studies evaluated weight changes in patients (Table 3). Thirteen case series and case reports also reported 49 children experiencing weight gain (Table 4). Weight was measured in patients with idiopathic thrombocytopenic purpura (ITP) in two RCTs [17][18], three case series [72–74] and one case report [101]. The majority of patients had significant weight increase of 6% to 10% during oral corticosteroid treatment.

**Table 4:** ADRs from case series and case reports.

Adverse effects	No. of pt with ADRs¶	No. of studies reporting ADR
**Cushingoid features**	**120**	**21**
**Behavioural changes**	**105**	**10**
**Increased appetite**	**83**	**4**
**Weight gain**	**49**	**13**
**Hypertension**	**36**	**8**
**Infection**	**29**	**10**
**Growth retardation**	**27**	**10**
**Gastrointestinal upset**	**23**	**4**
**Hyperglycaemia**	**22**	**4**
**Fatigue**	**21**	**3**
**Hypokalaemia**	**19**	**1**
**Myopathy**	**17**	**4**
**Insomnia**	**16**	**3**
**Headache**	**15**	**4**
**Cataracts**	**14**	**3**
**Gastric wall abnormalities**	**11**	**6**
**Osteoporosis**	**10**	**6**
**Dizziness**	**8**	**2**
**Oedema**	**8**	**2**
**Acne**	**7**	**4**
**Skin striae**	**7**	**3**
**Hirsutism**	**5**	**4**
**HPA axis suppression**	**2**	**2**
**Fever**	**2**	**2**
**Pigmentation**	**1**	**1**
**Rash**	**1**	**1**
**Increased intraocular pressure**	**1**	**1**
**Pancreatitis**	**1**	**1**
**Obstructive sleep apnoea**	**1**	**1**
**Oral Ulceration**	**1**	**1**
**Hypocalcaemia**	**1**	**1**
**Unknown**	**11**	**1**
**Total**	**674**	**47**

^¶^Some patients had more than one adverse drug reaction

Thirty one different ADRs were reported from case series and case reports with a total of 674 ADRs in 397 patients (Table 4). [67–87] [88–113].

In total, 43 patients died during treatment with oral corticosteroids (Table 5). Sixteen cohort studies and six RCTs reported that 278 children experienced infection during treatment periods (incidence 8.7%). Five studies reported 21 patients who died due to infection [28][63][89][96][106]. Infection was the commonest cause of death, accounting for half of the deaths. The majority of the children (nine patients) died after admission to intensive care with varicella zoster virus. Seven of these patients received oral prednisolone [28][63][89][96], and two leukaemic patients received oral dexamethasone [28][106]. Another two children with Kearns-Sayre syndrome died from hyperglycaemia after receiving oral prednisolone [92]. Twenty children (six prednisolone, 14 dexamethasone) with leukaemia died due to unknown reasons [27]. (Table 5).

**Table 5:** ADRs that led to death or drug discontinuation.

Causes	Discontinue	Death
**Infection**	**2**	**21**
**Hyperglycaemia**	**-**	**2**
**Behaviour changes**	**9**	**-**
**Growth retardation**	**4**	**-**
**Cushingoid features**	**4**	**-**
**Hypertension**	**2**	**-**
**Ulceration**	**2**	**-**
**HPA axis suppression**	**1**	**-**
**Vomiting**	**1**	**-**
**Increased intraocular pressure**	**1**	**-**
**Obstructive sleep apnoea**	**1**	**-**
**Unknown ADRs**	**65**	**20**
**Total**	**92**	**43**

Behavioural changes were one of the most common causes for medication withdrawal, reported in three studies with nine leukaemia patients, all of whom were treated with oral dexamethasone [27][28][98]. Additionally growth retardation in four infants with haemangioma led to discontinuation of oral prednisolone [32]. (Table 5).

## Discussion

Forty three patients died during treatment with oral corticosteroids. Infection was the most serious ADR and was responsible for half of the deaths. The incidence of infection with short course oral corticosteroids in children has previously been reported as (0.9%) [2]. It was significantly higher (8.7%) with long course treatment (P <0.0001). It is already well known that the immunosuppressant effects of corticosteroids in supra-physiological doses can lead to patients having increased susceptibility to infection, to deterioration in those with existing infections or to activation of latent infection [135]. To compound this their anti-inflammatory effects may mask symptoms and allow infections to progress significantly before detection. We believe this is the first study to quantify this greatly increased incidence of infection in patients on long-course rather than short-course oral corticosteroids.

Hyperglycaemia was another serious side effect associated with long courses of oral corticosteroids. There were no differences between the incidence of this ADR during short and long course treatment [2]. The risk during short course corticosteroids however was transient and resolved shortly after discontinuing the medication without intervention, however during long course treatment some patients needed daily insulin [27][28][90].

Behavioural change was twice as common during long course treatment (8.1%) compared with short course (4.7%) [2] and this difference was significant (P <0.0001). Treatment withdrawal and medication intervention were often required with long course treatment. Psychiatric ADRs ranging from insomnia to mood change and dementia have also been reported in adult patients [136][137]. The incidence of behavioural change in adults was reported as 5.7% in a review written in 1983 [138]. Our study suggests that behavioural problems may be more common in children.

The risk of HPA axis suppression during long course corticosteroids was greater with dexamethasone than prednisolone in leukaemia patients [29,51–58]. Also suppression was greater with oral corticosteroids (66% of patients) than inhaled corticosteroids (31% of patients) in asthmatic patients [66]. The suppression reported during the long course corticosteroids studies was severe and led to adrenal crisis. Fourteen patients with HPA axis suppression during long course treatment needed months to regain adequate adrenal function [36][57][58]. On the other hand, all the children with HPA axis suppression during short course treatment returned to a normal level of endogenous cortisol secretion within 10–12 days after discontinuation of the corticosteroids [2].

The three most commonly observed ADRs associated with long-course oral corticosteroids were weight gain, growth retardation and cushingoid features.

Weight gain was the most common side effect. This was not however significantly different to that reported for short-course corticosteroid treatment [2]. The risk of this ADR was greater with prednisolone than budesonide in children [26]. In adult patients weight gain was associated with longer duration of corticosteroids use, even with low-dose [139].

Growth retardation was the second most frequently observed side effect. The risk of this ADR was significantly greater with prednisolone than intravenous methylprednisolone [22]. Also the risk was significantly greater with boys than girls [43]. Comparing oral corticosteroids with inhaled corticosteroids (ICS), one study found that inhaled medication is significantly better than oral in terms of growth retardation [140]. This ADR has not been reported during short-course corticosteroid treatment. [2]

Cushingoid features were the third most frequently observed side effect caused by oral corticosteroids. The risk of this ADR was significantly greater with prednisolone than budesonide [14][15] (Fig 5). This ADR was not reported during short-course corticosteroids treatment [2]. In adult patients Cushingoid features were associated with longer duration of corticosteroids use, even with low-dose [139].

Hypertension was another side effect associated with long courses of oral corticosteroids occurring in 5.6% patients. This ADR was severe in some patients and occasionally needed medical intervention. Two infants stopped their medication because of this ADR and another eight required antihypertensive medication [22][38][70][109].

The prevalence of overall ADRs associated with long course corticosteroid use was high, and this finding may be underappreciated by clinicians. Because parents and patients may be concerned about the potential ADRs of corticosteroids prior to use, physicians need to be well apprised of their relative risks.

In conclusion this systematic review showed that the most serious ADR associated with long-course oral corticosteroids was infection reported in 9% patients and resulting in 21 deaths. The most frequent ADRs were weight gain, growth retardation and cushingoid features. Behavioural changes were the main reason for treatment discontinuation.

### Registration number

CRD42014010501

By PROSPERO International prospective register of systematic reviews

## Supporting Information

S1 Appendix Summary of included studies. (DOCX)

S2 Appendix PRISMA Checklist. (DOCX)
