# Supplementary material for: Systematic Review of the Toxicity of Long-Course Oral Corticosteroids in Children
Source: PLoS One. 2017 Jan 26;12(1):e0170259. doi: 10.1371/journal.pone.0170259 (PMC5268779; doi:10.1371/journal.pone.0170259)
Supplement: S1 Appendix — (DOCX) [file pone.0170259.s001.docx]

**Summary of included studies**

**Table 1 Summary of ADRs for leukaemia Patients**

| **Types of studies** | **Authors and**  **years** | **Country** | **Drug name** | **Duration** | **No. of patients** | **Age** | **ADRs detect method** | **No. of pt. withdraw** | **Mortality** | **Side effects**  **(P-value) ‡** | **No. of SE** | |
| --- | --- | --- | --- | --- | --- | --- | --- | --- | --- | --- | --- | --- |
|  |  |  |  |  |  |  |  |  |  |  | **A** | **B** |
| **RCTs** | Bostrom et al. 2003 (1) | USA | 1. Prednisolone | 7 Y | 530 | 1 – 18 Y | 1. Clinical assessment 2. Laboratory test 3. Glucose measured | 0 | 8 (7 infection, 1 varicella) | **Myopathy (0.0001)** | **8** | **34** |
|  |  |  | 1. Dexamethasone |  | 530 |  |  | 4 (behaviour) changed to prednisolone | 6 (5 infection, 1 varicella) | **Hyperglycaemia (0.01)** | **8** | **26** |
|  |  |  |  |  |  |  |  |  |  | **Virus infection** | **14** | **11** |
|  |  |  |  |  |  |  |  |  |  | **Infection** | **11** | **6** |
|  |  |  |  |  |  |  |  |  |  | **Pancreatitis (0.09)** | **2** | **7** |
|  |  |  |  |  |  |  |  |  |  | **Behaviour disturbance** | **0** | **4** |
|  |  |  |  |  |  |  |  |  |  | **Avascular necrosis** | **2** | **1** |
|  | Mitchell et al. 2005 (2) | UK | 1. Prednisolone | 5 Y | 805 | 1 – 10 Y | 1. Clinical assessment 2. Laboratory test 3. Weighted measured 4. Growth measured 5. Blood pressure measured 6. Glucose measured | 0 | 6 | **Behaviour disturbance** | **11** | **47** |
|  |  |  | 1. Dexamethasone |  | 798 |  |  | 47 (side effects) changed to prednisolone  3 (behaviour) changed to prednisolone | 14 | **Myopathy** | **4** | **22** |
|  |  |  |  |  |  |  |  |  |  | **Hyperglycaemia** | **12** | **13** |
|  |  |  |  |  |  |  |  |  |  | **Avascular necrosis** | **9** | **6** |
|  |  |  |  |  |  |  |  |  |  | **Hypertension** | **4** | **6** |
|  |  |  |  |  |  |  |  |  |  | **Weight gain** | **1** | **7** |
|  |  |  |  |  |  |  |  |  |  | **Osteoporosis** | **1** | **7** |
|  |  |  |  |  |  |  |  |  |  | **Other** | **1** | **7** |
|  | Einaudi et al. 2008 (3) | Italy | 1. Prednisolone | 30 D | 40 | 1.2 – 17.6 Y | HPA axis function (ACTH test) | 0 | 0 | **HPA axis suppression** | **24** | **16** |
|  |  |  | 1. Dexamethasone |  | 24 | 1.2 – 12.1 Y |  | 0 | 0 |  |  |  |
| **PCS** | Lightner et al 1981 (4) | USA | Prednisone | 30 D | 13 | 20 M – 14 Y | 1. Clinical assessment 2. HPA axis function | 0 | 0 | **HPA axis suppression** | **6** | **-** |
|  |  |  |  |  |  |  |  |  |  | **Infection** | **5** | **-** |
|  | Drigan et al 1992 (5) | USA | Prednisone | NA | 38 | 2 – 9 Y | Evaluated the behaviour | 0 | 0 | **Behaviour change** | **38** | **-** |
|  |  |  |  |  |  |  |  |  |  | **Insomnia** | **38** | **-** |
|  | Felner et al. 2000 (6) | USA | Dexamethasone | 4 W | 10 | 2 – 9.9 Y | 1. Clinical assessment 2. Weighted measured 3. HPA axis function (ACTH test) 4. Growth measured | 0 | 0 | **Weight gain** | **10** | **-** |
|  |  |  |  |  |  |  |  |  |  | **Myopathy** | **10** | **-** |
|  |  |  |  |  |  |  |  |  |  | **Fatigue** | **10** | **-** |
|  |  |  |  |  |  |  |  |  |  | **Abdominal pain** | **10** | **-** |
|  |  |  |  |  |  |  |  |  |  | **Diarrheal** | **10** | **-** |
|  |  |  |  |  |  |  |  |  |  | **Gastrointestinal upset** | **10** | **-** |
|  |  |  |  |  |  |  |  |  |  | **HPA axis suppression** | **3** | **-** |
|  |  |  |  |  |  |  |  |  |  | **Infection** | **1** | **-** |
|  | Kuperman et al. 2001 (7) | Brazil | Dexamethasone | 42 D | 15 | 1.5 – 12 Y | 1. Clinical assessment 2. Weighted measured 3. HPA axis function (ACTH test) | 0 | 0 | **HPA axis suppression** | **15** | **-** |
|  |  |  |  |  |  |  |  |  |  | **Weight gain** | **15** |  |
|  |  |  |  |  |  |  |  |  |  | **Increased appetite** | **15** | **-** |
|  |  |  |  |  |  |  |  |  |  | **Diarrheal** | **4** | **-** |
|  |  |  |  |  |  |  |  |  |  | **Gastrointestinal upset** | **4** | **-** |
|  |  |  |  |  |  |  |  |  |  | **Infection** | **4** | **-** |
|  | Petersen et al. 2003 (8) | Denmark | 1. Prednisone | 5 W | 10 | 2 – 15 Y | HPA axis function (ACTH test) | 0 | 0 | **HPA axis suppression** | **10** | **7** |
|  |  |  | 1. Dexamethasone | 3 W | 7 |  |  |  |  |  |  |  |

**Abbreviation:** ‡ P-value is given where these were provided in the relevant study. ACTH, Adrenocorticotropic hormone. RCTs, Randomise control trials. PCS, Prospective cohort studies.

**Table 1 Summary of ADRs for leukaemia Patients (continued)**

| **Types of studies** | **Authors and**  **years** | **Country** | **Drug name** | **Duration** | **No. of patients** | **Age** | **ADRs detect method** | **No. of pt. withdraw** | **Mortality** | **Side effects** | **No. of SE** | |
| --- | --- | --- | --- | --- | --- | --- | --- | --- | --- | --- | --- | --- |
|  |  |  |  |  |  |  |  |  |  |  | **A** | **B** |
| **PCS** | Mahachoklerwattana et al. 2004 (9) | Thailand | Prednisone | 28 D | 24 | 1 – 14 Y | 1. Clinical assessment 2. HPA axis function (ACTH test) | 0 | 0 | **HPA axis suppression** | **11** | **-** |
|  |  |  |  |  |  |  |  |  |  | **Infection** | **1** | **-** |
|  | Cunha et al. 2004 (10) | Brazil | Dexamethasone | 4 W | 28 | 1 – 14 Y | 1. Clinical assessment 2. HPA axis function (ACTH test) | 0 | 0 | **HPA axis suppression** | **28** | **-** |
|  |  |  |  |  |  |  |  |  |  | **Cushingoid features** | **20** | **-** |
|  |  |  |  |  |  |  |  |  |  | **Infection** | **11** | **-** |
|  | Rix et al. 2005 (11) | Denmark | Prednisone | 5 W | 17 | 1.8 – 14.6 Y | 1. Clinical assessment 2. HPA axis function (ACTH test) | 0 | 0 | **Infection** | **10** | **-** |
|  |  |  |  |  |  |  |  |  |  | **HPA axis suppression** | **8** | **-** |
|  | Gordijn et al. 2012 (12) | Netherlands | Dexamethasone | 4 W | 42 | 10.6 ± 3.9 Y | 1. Clinical assessment 2. HPA axis function (ACTH test) | 0 | 0 | **HPA axis suppression** | **42** | **-** |
|  |  |  |  |  |  |  |  |  |  | **Fatigue** | **42** | **-** |
|  |  |  |  |  |  |  |  |  |  | **Behaviour change** | **42** | **-** |
|  |  |  |  |  |  |  |  |  |  | **Insomnia** | **42** | **-** |
| **CS** | Elliott et al. 1985 (13) | UK | Prednisone | 2 – 10.5 M | 37 | 8 – 22 Y | - | 0 | 0 | **Cataract** | **12** | **-** |
| **CR** | Matsuzaki et al. 2008 (14) | Japan | Dexamethasone | 2 W | 1 | 5 Y | - | 0 | 1 (Infection) | **Infection (varicella)** | **1** | **-** |
|  | Watanabe et al. 1994 (15) | USA | Dexamethasone | 4 W | 2 | 13 + 17 Y | - | 2  (Behaviour) | 0 | **Behaviour change** | **2** | **-** |
|  |  |  |  |  |  |  |  |  |  | **Sleep disturbance** | **1** | **-** |
|  |  |  |  |  |  |  |  |  |  | **Fever** | **1** | **-** |

**Abbreviation:** ACTH, Adrenocorticotropic hormone. PCS, Prospective cohort studies. Cs, Case series. CR, Case report.

**Table 2 Summary of ADRs for Haemangioma Patients**

| **Types of studies** | **Authors and**  **years** | **Country** | **Drug name** | **Duration** | **No. of patients** | **Age** | **ADRs detect method** | **No. of pt. withdraw** | **Side effects**  **(P-value) ‡** | **No. of SE** | |
| --- | --- | --- | --- | --- | --- | --- | --- | --- | --- | --- | --- |
|  |  |  |  |  |  |  |  |  |  | **A** | **B** |
| **RCTs** | Elena Pope et al. 2007 (16) | Canada | 1. Prednisolone | 3 M | 10 | 1 – 4 M | 1. Clinical assessment 2. Laboratory test 3. Weighted measured 4. Growth measured 5. Blood pressure measured 6. HPA axis function 7. Glucose measured | 1 (vomiting) | **Weight gain** | **10** | **10** |
|  |  |  | 1. Methylprednisolone IV |  | 10 |  |  | 0 | **Hyperglycaemia** | **10** | **10** |
|  |  |  |  |  |  |  |  |  | **Growth retardation (0.001)** | **10** | **0** |
|  |  |  |  |  |  |  |  |  | **HPA axis suppression** | **8** | **6** |
|  |  |  |  |  |  |  |  |  | **Behaviour disturbance** | **3** | **3** |
|  |  |  |  |  |  |  |  |  | **Insomnia** | **3** | **1** |
|  |  |  |  |  |  |  |  |  | **Hypertension** | **2** | **1** |
|  |  |  |  |  |  |  |  |  | **Abdominal pain** | **1** | **1** |
|  |  |  |  |  |  |  |  |  | **Vomiting** | **1** | **1** |
|  |  |  |  |  |  |  |  |  | **Infection (varicella)** | **1** | **0** |
|  | Bauman et al. 2014 (17) | USA | 1. Prednisolone | 6 M | 8 | 2 W – 6 M | 1. Clinical assessment 2. Weighted measured 3. Growth measured 4. Blood pressure measured 5. HPA axis function 6. Glucose measured | 4 (growth retardation)  1 (Cushingoid features) | **Hypotension** | **0** | **11** |
|  |  |  |  |  | 11 |  |  |  | **Hypertension** | **8** | **0** |
|  |  |  |  |  |  |  |  |  | **Growth retardation** | **6** | **0** |
|  |  |  |  |  |  |  |  |  | **Weight loss** | **6** | **0** |
|  |  |  | 1. Propranolol |  |  |  |  | 0 | **HPA axis suppression** | **1** | **0** |
|  |  |  |  |  |  |  |  |  | **Infection** | **1** | **0** |
|  |  |  |  |  |  |  |  |  | **Cushingoid features** | **1** | **0** |
|  |  |  |  |  |  |  |  |  | **Hypoglycaemia** | **0** | **1** |
|  | Aly et al. 2015 (18) | Egypt | 1. Propranolol + Prednisolone | 6 M + 3 W | 20 | 2 – 8 M | 1. Clinical assessment 2. Laboratory test 3. Weighted measured 4. Growth measured 5. Blood pressure measured 6. Glucose measured | 0 | **Infection** | **2** | **0** |
|  |  |  |  |  |  |  |  |  | **Sleep disturbance** | **0** | **1** |
|  |  |  | 1. Propranolol | 6 M | 20 | 1 – 8 M |  |  |  |  |  |
| **PCS** | Boon et al. 1999 (19) | USA | Prednisolone | 2 – 21 M | 62 | 3 – 12 M | 1. Clinical assessment 2. Weighted measured 3. Growth measured 4. Blood pressure measured | 0 | **Cushingoid features** | **44** | **-** |
|  |  |  |  |  |  |  |  |  | **Weight loss** | **26** | **-** |
|  |  |  |  |  |  |  |  |  | **Growth retardation** | **22** | **-** |
|  |  |  |  |  |  |  |  |  | **Behaviour disturbance** | **18** | **-** |
|  |  |  |  |  |  |  |  |  | **Gastric wall abnormalities** | **13** | **-** |
|  |  |  |  |  |  |  |  |  | **Insomnia** | **8** | **-** |
|  |  |  |  |  |  |  |  |  | **Infection** | **4** | **-** |
|  |  |  |  |  |  |  |  |  | **Hypertension** | **1** | **-** |
|  |  |  |  |  |  |  |  |  | **Myopathy** | **1** | **-** |

**Abbreviation:** ‡ P-value is given where these were provided in the relevant study. RCTs, Randomise control trials. PCS, prospective cohort studies.

**Table 2 Summary of ADRs for Haemangioma Patients (continued)**

| **Types of studies** | **Authors and**  **years** | **Country** | **Drug name** | **Duration** | **No. of patients** | **Age** | **ADRs detect method** | **No. of pt. withdraw** | **Side effects** | **No. of SE** | |
| --- | --- | --- | --- | --- | --- | --- | --- | --- | --- | --- | --- |
|  |  |  |  |  |  |  |  |  |  | **A** | **B** |
| **PCS** | Pandey et al. 2009 (20) | India | 1. Prednisolone | NA | 499 | 1 – 49 M | 1. Clinical assessment 2. Growth measured 3. Blood pressure measured | 0 | **Gastric wall abnormalities** | **13** | **130** |
|  |  |  | 1. Prednisolone + Triamcinolone injection |  | 628 |  |  |  | **Skin atrophy** | **16** | **106** |
|  |  |  |  |  |  |  |  |  | **Hypopigmentation** | **7** | **101** |
|  |  |  |  |  |  |  |  |  | **Infection** | **55** | **91** |
|  |  |  |  |  |  |  |  |  | **Growth retardation** | **21** | **37** |
|  |  |  |  |  |  |  |  |  | **Cushingoid features** | **21** | **37** |
|  |  |  |  |  |  |  |  |  | **Hypertension** | **20** | **30** |
|  | Jalil et al. 2011 (21) | UK | Prednisolone | 3 – 4 M | 6 | 2 – 3 Y | 1. Clinical assessment 2. HPA axis function | 1 (adrenal suppression)  1 (pneumonia) | **HPA axis suppression** | **1** | **-** |
|  |  |  |  |  |  |  |  |  | **Infection** | **1** | **-** |
|  | Bertrand et al. 2011 (22) | Canada | Prednisone | 5 – 28 M | 12 | 1 – 9 M | 1. Clinical assessment 2. Growth measured 3. Blood pressure measured |  | **Infection** | **2** | **-** |
|  |  |  |  |  |  |  |  |  | **Behaviour disturbance** | **2** | **-** |
|  |  |  |  |  |  |  |  |  | **Growth retardation** | **1** | **-** |
|  |  |  |  |  |  |  |  |  | **Hypertension** | **1** | **-** |
|  |  |  |  |  |  |  |  |  | **Insomnia** | **1** | **-** |
|  | Grover et al. 2011 (23) | India | Prednisolone | 3 – 15 W | 20 | 0.5 – 12 M | 1. Clinical assessment 2. Weighted measured 3. Growth measured 4. Blood pressure measured | 1 (hypertension) | **Weight gain** | **3** | **-** |
|  |  |  |  |  |  |  |  |  | **Cushingoid features** | **2** | **-** |
|  |  |  |  |  |  |  |  |  | **Hypertension** | **1** | **-** |
|  | Price et al. 2011 (24) | USA | Prednisolone | 5.2 M | 42 | 4.5 M | 1. Clinical assessment 2. Weighted measured 3. Laboratory test 4. Growth measured 5. Blood pressure measured 6. Glucose measured | 1 (ulceration) | **Cushingoid features** | **42** | **-** |
|  |  |  |  |  |  |  |  |  | **Gastric wall abnormalities** | **4** | **-** |
|  |  |  |  |  |  |  |  |  | **Infection** | **3** | **-** |
|  |  |  |  |  |  |  |  |  | **Hypertension** | **2** | **-** |
|  |  |  |  |  |  |  |  |  | **Ulceration** | **1** | **-** |
|  |  |  |  |  |  |  |  |  | **Skin atrophy** | **1** | **-** |
|  |  |  |  |  |  |  |  |  | **hirsutism** | **1** | **-** |
|  |  |  |  |  |  |  |  |  | **Hypercholesterolemia** | **1** | **-** |
|  |  |  |  |  |  |  |  |  | **Weight loss** | **1** | **-** |
| **CS** | Enjolras et al. 1990 (25) | France | Prednisolone | 1 –18 M | 25 | 1 –7 M | - | 1 (Duodenal ulcer) | **Growth retardation** | **15** | **-** |
|  | Sadan and Wolach 1996 (26) | Israel | Prednisolone | 6 –12 W | 60 | 1 –13 M | - | 0 | **Behaviour change** | **60** | **-** |
|  |  |  |  |  |  |  |  |  | **Increased appetite** | **60** | **-** |
|  |  |  |  |  |  |  |  |  | **Cushingoid features** | **32** | **-** |
|  |  |  |  |  |  |  |  |  | **Growth retardation** | **2** | **-** |
|  |  |  |  |  |  |  |  |  | **Osteoporosis** | **1** | **-** |
|  | Uysal et al. 2001 (27) | Turkey | Methylprednisolone | 4 W | 15 | 1 – 23 M | - | 0 | **Increased appetite** | **15** | **-** |
|  |  |  |  |  |  |  |  |  | **Cushingoid features** | **10** | **-** |
|  |  |  |  |  |  |  |  |  | **Infection** | **3** | **-** |
|  |  |  |  |  |  |  |  |  | **Gastroenteritis** | **2** | **-** |
|  |  |  |  |  |  |  |  |  | **Growth retardation** | **1** | **-** |

**Abbreviation:** PCS, prospective cohort studies. Cs, case series.

**Table 2 Summary of ADRs for Haemangioma Patients (continued)**

| **Types of studies** | **Authors and**  **years** | **Country** | **Drug name** | **Duration** | **No. of patients** | **Age** | **ADRs detect method** | **No. of pt. withdraw** | **Side effects** | **No. of SE** | |
| --- | --- | --- | --- | --- | --- | --- | --- | --- | --- | --- | --- |
|  |  |  |  |  |  |  |  |  |  | **A** | **B** |
| **CS** | Mallo et al. 2004 (28) | Spain | Prednisolone | 6 M | 8 | 4 – 24 M | - | 0 | **Cushingoid features** | **1** | **-** |
|  |  |  |  |  |  |  |  |  | **Growth retardation** | **1** | **-** |
|  |  |  |  |  |  |  |  |  | **Hirsutism** | **1** | **-** |
|  | Corapcioglu et al. 2011 (29) | Turkey | Prednisolone | 4 W | 12 | 1 – 24 M | - | 10 (Lack of response)  1 (Cuchingoid) | **Ulceration** | **5** | **-** |
|  |  |  |  |  |  |  |  |  | **Cushingoid features** | **1** | **-** |
|  | Nieuwenhuis et al. 2013 (30) | Netherlands | Prednisolone | 3 – 4 W | 21 | 5 – 14 M | - | 0 | **Cushingoid features** | **8** | **-** |
|  |  |  |  |  |  |  |  |  | **Increased appetite** | **5** | **-** |
|  |  |  |  |  |  |  |  |  | **Behaviour change** | **4** | **-** |
|  |  |  |  |  |  |  |  |  | **Hypertension** | **2** | **-** |
|  |  |  |  |  |  |  |  |  | **HPA axis suppression** | **1** | **-** |
|  |  |  |  |  |  |  |  |  | **Acne** | **1** | **-** |
| **CR** | Hernandez et al. 2007 (31) | Spain | Prednisolone | 22 M | 1 | 8 M | - | 1 (obstructive sleep apnoea) | **Obstructive sleep apnoea** | **1** | **-** |
|  |  |  |  |  |  |  |  |  | **Cushingoid features** | **1** | **-** |
|  |  |  |  |  |  |  |  |  | **Behaviour change** | **1** | **-** |
|  |  |  |  |  |  |  |  |  | **Sleep disturbance** | **1** | **-** |
|  | Koay et al. 2011 (32) | Malaysia | Prednisolone | 1 M | 1 | 3 M | - | 0 | **Weight gain** | **1** | **-** |
|  | Morais et al. 2011 (33) | Portugal | Prednisolone | 12 M | 1 | 2 M | - | 1 (Hypertension) | **Hypertension** | **1** | **-** |

**Abbreviation:** Cs, case series. CR, Case report.

**Table 3 Summary of ADRs for Asthmatic Patients**

| **Types of studies** | **Authors and**  **years** | **Country** | **Drug name** | **Duration** | **No. of patients** | **Age** | **ADRs detect method** | **No. of pt. withdraw** | **Side effects**  **(P-value) ‡** | **No. of SE** | |
| --- | --- | --- | --- | --- | --- | --- | --- | --- | --- | --- | --- |
|  |  |  |  |  |  |  |  |  |  | **A** | **B** |
| **RCTs** | Wolthers et al. 1990 (34) | Denmark | 1. Prednisolone | 30 D | 14 | 7 – 11 Y | 1. Clinical assessment 2. Growth measured | 1 (Infection) | **Growth retardation** | **12** | **0** |
|  |  |  | 1. Placebo |  |  |  |  | 0 | **Infection (varicella)** | **1** | **0** |
|  |  |  |  |  |  |  |  |  | **Acne** | **1** | **0** |
|  |  |  |  |  |  |  |  |  | **Headache** | **1** | **0** |
|  |  |  |  |  |  |  |  |  | **Behaviour disturbance** | **1** | **0** |
| **PCS** | Rooklin et al 1979 (35) | USA | Prednisolone | 1. **˂** 1 Y | 10 | 4 – 18 Y | 1. Clinical assessment 2. Growth measured | 0 | **Growth retardation** | **7** | **14** |
|  |  |  |  | 1. **>**  1 Y | 14 |  |  |  | **Cataract** | **0** | **7** |
|  | Wiersbitzky et al 1982 (36) | Germany | Prednisolone | 18 M | 29 | 3 – 17 Y | Growth measured | 0 | **Growth retardation** | **17** | **-** |
|  | Nassif et al. 1987 (37) | USA | Prednisolone | 1.3 ± 1 Y (minimum 6 months) | 24 | 7 – 13 Y | 1. Clinical assessment 2. HPA axis function 3. Weighted measured 4. Growth measured | 0 | **Growth retardation** | **24** | **-** |
|  |  |  |  |  |  |  |  |  | **Weight gain** | **24** | **-** |
|  |  |  |  |  |  |  |  |  | **HPA axis suppression** | **8** | **-** |
|  |  |  |  |  |  |  |  |  | **Infection** | **3** | **-** |
|  |  |  |  |  |  |  |  |  | **Cataract** | **1** | **-** |
|  | kelly et al. 2008 (38) | USA | Prednisolone | NA | 877 | 5 – 12 Y | Bone mineral density measurements | 0 | **Decrease bone density** | **157** | **-** |
|  |  |  |  |  |  |  |  |  | **Osteoporosis** | **4** | **-** |
|  | Hawcutt et al. 2015 (39) | UK | 1. Prednisolone | NA | 27 | 5 – 18 Y | HPA axis function (ACTH test) | 0 | **HPA axis suppression (0.0008)** | **18** | **91** |
|  |  |  | 1. Inhaled corticosteroid |  | 289 |  |  |  |  |  |  |
| **CS** | Sanders et al. 1992 (40) | USA | 1. Prednisolone | 19 M | 3 | 9 – 16 Y | - | 0 | **Hypertension** | **3** | **6** |
|  |  |  | 1. Methylprednisolone |  | 6 | 8.5 – 15 Y |  |  | **Headache** | **3** | **5** |
|  |  |  |  |  |  |  |  |  | **Gastrointestinal upset** | **1** | **0** |
| **CR** | Silk et al 1988 (41) | USA | Prednisolone | 3 W | 1 | 16 Y | - | 1 (Died) | **Infection (varicella)** | **1** | **-** |
|  | Zychowicz and Wicinska 1982 (42) | Poland | Prednisolone | 1 – 3 M | 2 | 9 + 16 Y | - | 0 | **Growth retardation** | **2** | **-** |
|  | Abuekteish et al. 1995 (43) | UK | Prednisolone | 5 M | 1 | 13 Y | - | 0 | **Cataract** | **1** | **-** |
|  |  |  |  |  |  |  |  |  | **Behaviour change** | **1** | **-** |

**Abbreviation:** ‡ P-value is given where these were provided in the relevant study. RCTs, Randomise control trials. PCS, Prospective cohort studies. Cs, Case series. CR, Case report.

**Table 4 Summary of ADRs for Nephrotic Syndrome Patients**

| **Types of studies** | **Authors and**  **years** | **Country** | **Drug name** | **Duration** | **No. of patients** | **Age** | **ADRs detect method** | **No. of pt. withdraw** | **Mortality** | **Side effects** | **No. of SE** | |
| --- | --- | --- | --- | --- | --- | --- | --- | --- | --- | --- | --- | --- |
|  |  |  |  |  |  |  |  |  |  |  | **A** | **B** |
| **RCTs** | Broyer et al. 1997 (44) | France | 1. Prednisolone | 12 M | 20 | 8.5 ± 4 Y | 1. Clinical assessment 2. Laboratory test 3. Weighted measured 4. Growth measured 5. Blood pressure measured 6. Glucose measured | 0 | 0 | **Weight gain** | **20** | **20** |
|  |  |  | 1. Deflazacort |  | 20 | 9.2 ± 2.7 Y |  | 0 | 0 | **Decrease bone density** | **20** | **20** |
|  |  |  |  |  |  |  |  |  |  | **Cushingoid features** | **11** | **7** |
|  |  |  |  |  |  |  |  |  |  | **Hypertension** | **3** | **3** |
|  |  |  |  |  |  |  |  |  |  | **Gastric ulcer** | **0** | **2** |
|  |  |  |  |  |  |  |  |  |  | **Hematemesis** | **1** | **0** |
|  |  |  |  |  |  |  |  |  |  | **Osteoporosis** | **1** | **0** |
| **PCS** | Miltenyi et al. 1983 (45) | Hungary | Prednisolone | 6 – 72 M | 24 | 1.5 – 13.8 Y | 1. Clinical assessment 2. Blood pressure measured 3. Bone density measurements 4. Ophthalmologist | 0 | 0 | **Cushingoid features** | **15** | **-** |
|  |  |  |  |  |  |  |  |  |  | **Hypertension** | **13** | **-** |
|  |  |  |  |  |  |  |  |  |  | **Osteoporosis** | **6** | **-** |
|  |  |  |  |  |  |  |  |  |  | **Cataract** | **1** | **-** |
|  | Foote et al. 1985 (46) | UK | Prednisolone | 8 W | 80 | 5 – 24 Y | Growth measured | 0 | 0 | **Growth retardation** | **72** | **-** |
|  | Merritt et al. 1986 (47) | USA | Prednisone | 4 W | 23 | 1 – 14 Y | Weighted measured | 0 | 0 | **Weight gain** | **20** | **-** |
|  | Garin et al. 1986 (48) | USA | Prednisolone | 1 M | 9 | 10 – 20 Y | 1. Clinical assessment 2. Blood pressure measured | 0 | 0 | **Cushingoid features** | **9** | **-** |
|  |  |  |  |  |  |  |  |  |  | **Hypertension** | **9** | **-** |
|  |  |  |  |  |  |  |  |  |  | **Skin rash** | **4** | **-** |
|  | Nammalwar et al. 2006 (49) | India | Prednisolone | 12 M | 34 | Children | Clinical assessment | 0 | 0 | **Infection** | **22** | **-** |
|  |  |  |  |  |  |  |  |  |  | **Hair loss** | **2** | **-** |
|  | Bahat et al. 2007 (50) | Turkey | Prednisolone | 4 – 101 M | 8 | 3.7 – 14 Y | 1. Blood pressure measured 2. Bone density measurements | 0 | 0 | **Hypertension** | **2** | **-** |
|  |  |  |  |  |  |  |  |  |  | **Osteoporosis** | **2** | **-** |
|  | Upadhyay et al. 2016 (51) | India | Prednisolone | 12 W | 45 | 2 – 14 Y | Behaviour and sleep measured | 0 | 0 | **Behavioural change** | **45** | **-** |
|  |  |  |  |  |  |  |  |  |  | **Insomnia** | **45** | **-** |
| **CS** | Rizzonia et al. 1986 (52) | Italy | Prednisone | 7 – 81 M | 21 | 2 – 9 Y |  | 2  (Cushingoid features) |  | **Cushingoid features** | **7** | **-** |
|  |  |  |  |  |  |  |  |  |  | **Weight gain** | **4** | **-** |
|  |  |  |  |  |  |  |  |  |  | **Hypertension** | **2** | **-** |
|  |  |  |  |  |  |  |  |  |  | **Hypocalcaemia** | **1** | **-** |
| **CR** | Fujita et al. 1983 (53) | Japan | Prednisolone | 7 W | 1 | 14 Y | - | 0 | 1  (Infection) | **Infection (varicella)** | **1** | **-** |
|  | George et al. 1983 (54) | USA | Prednisolone | 1 Y | 1 | 13 Y | - | 0 | 0 | **Hyperglycaemia** | **1** | **-** |
|  |  |  |  |  |  |  |  |  |  | **Myopathy** | **1** | - |
|  | Kaneda et al. 1984 (55) | Japan | Prednisolone | 16 M | 1 | 11 Y | - | 0 | 0 | **Cushingoid features** | **1** | **-** |
|  |  |  |  |  |  |  |  |  |  | **Weight gain** | **1** | **-** |
|  |  |  |  |  |  |  |  |  |  | **Myopathy** | **1** | **-** |
|  |  |  |  |  |  |  |  |  |  | **Striae rubra** | **1** | **-** |
|  | Tsuruga et al. 2009 (56) | Japan | Prednisolone | 4 M | 1 | 11 Y | - | 0 | 0 | **Weight gain** | **1** | **-** |
|  |  |  |  |  |  |  |  |  |  | **Osteoporosis** | **1** | **-** |
|  |  |  |  |  |  |  |  |  |  | **Growth retardation** | **1** | **-** |

**Abbreviation:** RCTs, Randomise control trials. PCS, Prospective cohort studies. Cs, Case series. CR, Case report.

**Table 5 Summary of ADRs for Cystic Fibrosis Patients**

| **Types of studies** | **Authors and**  **years** | **Country** | **Drug name** | **Duration** | **No. of patients** | **Age** | **ADRs detect method** | **No. of pt.**  **withdraw** | **Side effects**  **(P-value) ‡** | **No. of SE** | | |
| --- | --- | --- | --- | --- | --- | --- | --- | --- | --- | --- | --- | --- |
|  |  |  |  |  |  |  |  |  |  | **A** | **B** | **C** |
| **RCTS** | Greally et al.  1994 (57) | UK | 1. Prednisolone | 24 M | 12 | 5.5 – 19.5 Y | 1. Clinical assessment 2. Laboratory test 3. Weighted measured 4. Blood pressure measured 5. Glucose measured | 0 | **Weight gain** | **12** | **0** | **-** |
|  |  |  | 1. Placebo |  | 12 |  |  | 0 | **Hyperglycaemia** | **1** | **0** | **-** |
|  | Einaudi et al.  1995 (58) | USA | 1. Prednisolone (1mg/kg/day) Daily | 24 M | 95 | 6 – 14 Y | 1. Clinical assessment 2. Laboratory test 3. Weighted measured 4. Growth measured 5. Glucose measured 6. Ophthalmologist | 0 | **Growth retardation (0.05)** | **24** | **31** | **11** |
|  |  |  | 1. Prednisolone (2mg/kg/day) Alternative Day |  | 95 |  |  | 0 | **Hyperglycaemia (0.05)** | **12** | **26** | **7** |
|  |  |  | 1. Placebo |  | 95 |  |  | 0 | **Cataracts** | **3** | **11** | **7** |

**Abbreviation:** ‡ P-value is given where these were provided in the relevant study. RCTs, Randomise control trials.

**Table 6 Summary of ADRs for Crohn's Disease Patients**

| **Types of studies** | **Authors and years** | **Country** | **Drug name** | **Duration** | **No. of patients** | **Age** | **ADRs detect method** | **No. of pt. Withdraw** | **Side effects**  **(P-value)‡** | **No. of SE** | |
| --- | --- | --- | --- | --- | --- | --- | --- | --- | --- | --- | --- |
|  |  |  |  |  |  |  |  |  |  | **A** | **B** |
| **RCTS** | Ruuska et al. 1994 (59) | Finland | 1. Prednisolone | 11 W | 9 | 8.5 – 18.6 Y | 1. Clinical assessment 2. Weighted measured | 1 (side effect) | **Weight gain** | **9** | **10** |
|  |  |  | 1. Enteral feeding |  | 11 |  |  | 1 (pain) |  |  |  |
|  | Levine et al. 2003 (60) | Israel | 1. Prednisone | 10 W | 14 | 8–18 Y | 1. Clinical assessment 2. Laboratory test 3. HPA axis function (ACTH test) 4. Weighted measured | 0 | **Weight gain (0.05)** | **14** | **19** |
|  |  |  | 1. Budesonide |  | 19 |  |  | 0 | **Cushingoid features (0.05)** | **10** | **6** |
|  |  |  |  |  |  |  |  |  | **Acne** | **4** | **3** |
|  |  |  |  |  |  |  |  |  | **Virus infection** | **2** | **0** |
|  |  |  |  |  |  |  |  |  | **HPA axis suppression** | **1** | **0** |
|  |  |  |  |  |  |  |  |  | **Insomnia** | **0** | **1** |
|  |  |  |  |  |  |  |  |  | **Hirsutism** | **4** | **2** |
|  |  |  |  |  |  |  |  |  | **Myopathy (0.07)** | **5** | **1** |
|  |  |  |  |  |  |  |  |  | **Headache** | **1** | **5** |
|  |  |  |  |  |  |  |  |  | **Fever** | **1** | **5** |
|  |  |  |  |  |  |  |  |  | **Behavioural changes** | **1** | **1** |
|  | Escher et al. 2004 (61) | USA | 1. Prednisone | 12 W | 26 | 6–16 Y | 1. Clinical assessment 2. HPA axis function (ACTH test) | 8 (side effect) | **HPA axis suppression** | **19** | **9** |
|  |  |  |  |  |  |  |  |  | **Cushingoid features (0.01)** | **16** | **5** |
|  |  |  | 1. Budesonide |  | 22 |  |  | 3 (side effect) | **Abdominal pain** | **6** | **3** |
|  |  |  |  |  |  |  |  |  | **Acne (0.033)** | **7** | **1** |
|  |  |  |  |  |  |  |  |  | **Headache** | **4** | **4** |
|  |  |  |  |  |  |  |  |  | **Vomiting** | **3** | **3** |
|  |  |  |  |  |  |  |  |  | **Myopathy** | **2** | **2** |
|  |  |  |  |  |  |  |  |  | **Fatigue** | **0** | **3** |
|  |  |  |  |  |  |  |  |  | **Fever** | **2** | **1** |
|  |  |  |  |  |  |  |  |  | **Diarrhoea** | **3** | **1** |
|  |  |  |  |  |  |  |  |  | **Nausea** | **2** | **1** |
|  |  |  |  |  |  |  |  |  | **Anorexia** | **1** | **2** |
|  |  |  |  |  |  |  |  |  | **Hirsutism** | **3** | **2** |
|  |  |  |  |  |  |  |  |  | **Behavioural changes** | **3** | **5** |
|  |  |  |  |  |  |  |  |  | **Insomnia** | **4** | **5** |
|  |  |  |  |  |  |  |  |  | **Hair loss** | **3** | **1** |
|  |  |  |  |  |  |  |  |  | **Peripheral oedema** | **1** | **0** |
|  |  |  |  |  |  |  |  |  | **Bruising easily** | **1** | **1** |
|  |  |  |  |  |  |  |  |  | **Skin striae** | **1** | **0** |
|  | Levine et al. 2009 (62) | Israel | 1. Budesonide 9mg/day | 10 W | 35 | 10-19 Y | 1. Clinical assessment 2. Laboratory test 3. HPA axis function (ACTH test) 4. Weighted measured | 0 | **Weight gain (0.05)** | **35** | **35** |
|  |  |  |  |  |  |  |  |  | **HPA axis suppression** | **15** | **17** |
|  |  |  | 1. Budesonide 12mg/day |  | 35 |  |  | 0 | **Acne** | **3** | **1** |
|  |  |  |  |  |  |  |  |  | **Hirsutism** | **0** | **1** |
|  |  |  |  |  |  |  |  |  | **Peripheral oedema** | **1** | **0** |
| **CR** | Wales and Milner 1988 (63) | UK | Prednisolone | 3 W | 1 | 11.6 Y | - | 0 | **Growth retardation** | **1** | **-** |
|  |  |  |  |  |  |  |  |  | **Weight gain** | **1** | **-** |

**Abbreviation:** ACTH, Adrenocorticotropic hormone. ‡ P-value is given where these were provided in the relevant study. RCTs, Randomise control trials. CR, Case report.

**Table 7 Summary of ADRs for Idiopathic Thrombocytopenic Purpura Patients**

| **Types of studies** | **Authors and years** | **Country** | **Drug name** | **Duration** | **No. of patients** | **Age** | **ADRs detect method** | **No. of pt. withdraw** | **Side effects**  **(P-value) ‡** | **No. of SE** | |
| --- | --- | --- | --- | --- | --- | --- | --- | --- | --- | --- | --- |
|  |  |  |  |  |  |  |  |  |  | **A** | **B** |
| **RCTS** | Imbach et al. 1985 (64) | Switzerland ‎ | 1. Prednisone | 21 D | 47 | Mean 6.3 Y | 1. Clinical assessment 2. Laboratory test 3. Weighted measured | 0 | **Weight gain** | **28** | **0** |
|  |  |  |  |  |  |  |  |  | **Cushingoid features** | **28** | **0** |
|  |  |  | 1. Immunoglobulin IV | 5 D | 47 | Mean 6.10 Y |  | 0 | **Acne** | **3** | **0** |
|  |  |  |  |  |  |  |  |  | **Headache** | **0** | **8** |
|  |  |  |  |  |  |  |  |  | **Fever** | **0** | **6** |
|  |  |  |  |  |  |  |  |  | **Dizziness** | **0** | **3** |
|  |  |  |  |  |  |  |  |  | **Vomiting** | **0** | **3** |
|  |  |  |  |  |  |  |  |  | **Other** | **3** | **0** |
|  | Blanchette et al. 1994 (65) | Canada | 1. Prednisone | 21 D | 39 | 6 M- 18 Y | 1. Clinical assessment 2. Laboratory test 3. Weighted measured | 0 | **Weight gain** | **39** | **0** |
|  |  |  | 1. Immunoglobulin IV | 2 D | 107 |  |  | 0 | **Headache** | **0** | **14** |
|  |  |  |  |  |  |  |  |  | **Fever** | **0** | **14** |
|  |  |  |  |  |  |  |  |  | **Vomiting** | **0** | **14** |
|  |  |  |  |  |  |  |  |  | **Dizziness** | **0** | **14** |
| **CS** | Chen et al. 1997(66) | Taiwan | Dexamethasone | 6 M | 7 | 5 –16 Y | - | 0 | **Myopathy** | **12** | - |
|  |  |  |  |  |  |  |  |  | **Weight gain** | **7** | - |
|  |  |  |  |  |  |  |  |  | **Dizziness** | **7** | - |
|  |  |  |  |  |  |  |  |  | **Acne** | **4** | - |
|  |  |  |  |  |  |  |  |  | **Fatigue** | **2** | - |
|  | Kuhne et al. 1997 (67) | Canada | Dexamethasone | 6 M | 11 | 2.5 –14.5 Y | - | 3 (Side effect) | **Weight gain (0.005)** | **11** | - |
|  |  |  |  |  |  |  |  |  | **Behaviour change** | **10** |  |
|  |  |  |  |  |  |  |  |  | **Fatigue** | **10** | - |
|  |  |  |  |  |  |  |  |  | **Facial flushing** | **7** | - |
|  |  |  |  |  |  |  |  |  | **Gastrointestinal upset** | **7** | - |
|  |  |  |  |  |  |  |  |  | **Headache** | **5** | - |
|  |  |  |  |  |  |  |  |  | **Sore throat** | **4** | - |
|  |  |  |  |  |  |  |  |  | **Increased appetite** | **3** | - |
|  |  |  |  |  |  |  |  |  | **Loss appetite** | **3** | - |
|  |  |  |  |  |  |  |  |  | **Pruritus** | **3** | - |
|  |  |  |  |  |  |  |  |  | **Myopathy** | **3** | - |
|  |  |  |  |  |  |  |  |  | **Dizziness** | **1** | - |
|  |  |  |  |  |  |  |  |  | **Gastroenteritis** | **1** | - |
|  |  |  |  |  |  |  |  |  | **Palmar erythema** | **1** | - |

**Abbreviation:** ‡ P-value is given where these were provided in the relevant study. RCTs, Randomise control trials. CS, Case series.

**Table 7 Summary of ADRs for Idiopathic Thrombocytopenic Purpura Patients (continued)**

| **Types of studies** | **Authors and years** | **Country** | **Drug name** | **Duration** | **No. of patients** | **Age** | **ADRs detect method** | **No. of pt. withdraw** | **Side effects** | **No. of SE** | |
| --- | --- | --- | --- | --- | --- | --- | --- | --- | --- | --- | --- |
|  |  |  |  |  |  |  |  |  |  | **A** | **B** |
| **CS** | Borgna-Pignatti et al. 1997 (68) | Italy | Dexamethasone | 6 M | 17 | 4 – 17 Y | - | 1 (Side effect)  1 (Lack of response) | **Weight gain** | **17** | - |
|  |  |  |  |  |  |  |  |  | **Fatigue** | **9** | - |
|  |  |  |  |  |  |  |  |  | **Behaviour change** | **8** | - |
|  |  |  |  |  |  |  |  |  | **Hirsutism** | **2** | - |
|  |  |  |  |  |  |  |  |  | **Gastrointestinal upset** | **2** | - |
|  |  |  |  |  |  |  |  |  | **Striae rubra** | **1** | - |
|  |  |  |  |  |  |  |  |  | **Headache** | **1** | - |
|  |  |  |  |  |  |  |  |  | **Acne** | **1** | - |
|  | Wali et al. 2002 (69) | Oman | Dexamethasone | 6 M | 13 | 2 – 14 Y | - | 0 | **Behaviour change** | **13** | - |
|  |  |  |  |  |  |  |  |  | **Sleep disturbance** | **13** | - |
|  |  |  |  |  |  |  |  |  | **Hyperglycaemia** | **13** | - |
|  |  |  |  |  |  |  |  |  | **Gastrointestinal upset** | **13** | - |
| **CR** | Sakurai et al. 1999 (70) | Japan | Prednisolone | 2 M | 2 | 5 M+ 5 Y | - | 0 | **Weight gain** | **1** | - |
|  |  |  |  |  |  |  |  |  | **Behaviour change** | **1** | - |

**Abbreviation:** CS, Case series. CR, Case report.

**Table 8 Summary of ADRs form Mixed Conditions**

| **Types of studies** | **Authors**  **and years** | **Country** | **Medical diagnosis** | **Drug name** | **Duration** | **No. of patients** | **Age** | **ADRs detect method** | **No. of pt. withdraw** | **Mortality** | **Side effects**  **(P-value) ‡** | **No. of SE** | |
| --- | --- | --- | --- | --- | --- | --- | --- | --- | --- | --- | --- | --- | --- |
|  |  |  |  |  |  |  |  |  |  |  |  | **A** | **B** |
| **RCTs** | Fenichel et  al. 1991 (71) | USA | Duchenne muscular dystrophy | 1. Prednisolone 0.75mg/kg/day (Daily) | 12 M | 33 | 5 – 15 Y | 1. Clinical assessment 2. Laboratory test 3. Weighted measured 4. Blood pressure measured 5. Blood glucose measured 6. Ophthalmologist | 2 (side effects) | 0 | **Weight gain** | **33** | **33** |
|  |  |  |  |  |  |  |  |  |  |  | **Hypertension** | **31** | **29** |
|  |  |  |  |  |  |  |  |  |  |  | **Cushingoid features** | **18** | **16** |
|  |  |  |  | 1. Prednisolone 1.5mg/kg/day (Alternate Day) |  | 33 |  |  | 0 | 0 | **Behaviour disturbance** | **15** | **14** |
|  |  |  |  |  |  |  |  |  |  |  | **Hirsutism** | **17** | **12** |
|  |  |  |  |  |  |  |  |  |  |  | **Gastrointestinal upset** | **13** | **10** |
|  |  |  |  |  |  |  |  |  |  |  | **Rash** | **9** | **7** |
|  |  |  |  |  |  |  |  |  |  |  | **Bruising easily** | **3** | **0** |
|  |  |  |  |  |  |  |  |  |  |  | **Glycosuria** | **2** | **1** |
|  |  |  |  |  |  |  |  |  |  |  | **Cataracts** | **2** | **0** |
|  | Zulian et al.  2011 (72) | Italy | Juvenile Localized Scleroderma | 1. Methotrexate + Prednisone | 3 M | 46 | 6 – 17 Y | 1. Clinical assessment 2. Laboratory test 3. Weighted measured | 0 | 0 | **Weight gain** | **5** | **10** |
|  |  |  |  | 1. Placebo + Prednisone |  | 24 |  |  | 0 | 0 | **Skin striae (0.02)** | **4** | **1** |
|  | Ozturk et al. 2011 (73) | Turkey | Chronic Rhinosinusitis | 1. Methylprednisolone | 1 M | 22 | 6 – 17 Y | 1. Clinical assessment 2. Weighted measured 3. Blood pressure measured | 0 | 0 | **Weight gain (0.08)** | **16** | **11** |
|  |  |  |  | 1. Placebo |  | 23 |  |  | 0 | 0 | **Increased appetite** | **16** | **11** |
|  | Zannolli et al. 2012 (74) | Italy | Ataxia telangiectasia | 1. Betamethasone | 1 M | 13 | 3 – 16 Y | 1. Clinical assessment 2. Weighted measured | 0 | 0 | **Weight gain** | **12** | **4** |
|  |  |  |  | 1. Placebo |  |  |  |  | 0 | 0 | **Cushingoid features** | **8** | **0** |
|  |  |  |  |  |  |  |  |  |  |  | **Behaviour disturbance** | **1** | **0** |
|  | Woynarowski et al. 2013 (75) | Poland | Autoimmune Hepatitis | 1. Prednisolone | 12 M | 27 | 11 – 17 Y | 1. Clinical assessment 2. Laboratory test 3. Weighted measured 4. Glucose measured | 0 | 0 | **Weight gain (0.006)** | **27** | **19** |
|  |  |  |  | 1. Budesonide |  | 19 | 9 – 17 Y |  | 0 | 0 | **Cushingoid features** | **12** | **2** |
|  |  |  |  |  |  |  |  |  |  |  | **Acne** | **7** | **4** |
|  |  |  |  |  |  |  |  |  |  |  | **Skin striae** | **3** | **1** |
| **PCS** | Dowell and Bresee. 1993 (76) | USA | Mixed | Prednisolone | 1 M | 14 | 5 – 18 Y | Clinical assessment | 0 | 4 (Infection) | **Infection (varicella)** | **14** | **-** |
|  | Connolly et al. 2002 (77) | USA | Duchenne Muscular Dystrophy | 1. Prednisone 5mg/kg/dose twice weekly | 12 - 32 M | 20 | 5.2 – 10.7 Y | 1. Clinical assessment 2. Blood pressure measured 3. Glucose measured 4. Weighted measured 5. Growth measured 6. Ophthalmologist | 0 | 0 | **Weight gain** | **11** | **4** |
|  |  |  |  | 1. Prednisone 0.75 mg/kg/day for 10 day each months | 7.7 ± 0.6 M | 4 | 7.3 ± 0.6 Y |  |  |  | **Increased appetite** | **7** | **4** |
|  |  |  |  |  |  |  |  |  |  |  | **Behaviour change** | **2** | **4** |
|  |  |  |  |  |  |  |  |  |  |  | **Insomnia** | **2** | **4** |
|  |  |  |  |  |  |  |  |  |  |  | **Growth retardation** | **0** | **4** |

**Abbreviation:** ‡ P-value is given where these were provided in the relevant study. RCTs, Randomise control trials. PCS, Prospective cohort studies.

**Table 8 Summary of ADRs form Mixed Conditions (continued)**

| **Types of studies** | **Authors**  **and years** | **Country** | **Medical diagnosis** | **Drug name** | **Duration** | **No. of patients** | **Age** | **ADRs detect method** | **No. of pt. withdraw** | **Mortality** | **Side effects** | **No. of SE** | |
| --- | --- | --- | --- | --- | --- | --- | --- | --- | --- | --- | --- | --- | --- |
|  |  |  |  |  |  |  |  |  |  |  |  | **A** | **B** |
| **PCS** | Ait Ourhoui et al. 2010 (78) | France | Alopecia Areata | Prednisolone | 3 – 6 M | 34 | 12±3 Y | Clinical assessment | 0 | 0 | **Fatigue** | **3** | **-** |
|  |  |  |  |  |  |  |  |  |  |  | **Infection** | **1** | **-** |
|  |  |  |  |  |  |  |  |  |  |  | **Gastric wall abnormalities** | **1** | **-** |
|  | Miura et al. 2011 (79) | Japan | Kawasaki Disease | Prednisolone | 2 – 6 W | 21 | 24 – 66 M | 1. Clinical assessment 2. Blood pressure measured 3. Glucose measured 4. Laboratory test | 0 | 0 | **Hypertension** | **11** | **-** |
|  |  |  |  |  |  |  |  |  |  |  | **Hyponatremia** | **3** | **-** |
|  |  |  |  |  |  |  |  |  |  |  | **Hypokalemia** | **1** | **-** |
|  |  |  |  |  |  |  |  |  |  |  | **Hyperglycemia** | **1** | **-** |
|  | Torok and Arkachaisri 2012 (80) | Singapore | Localized scleroderma | Prednisolone | 10 M | 36 | 4.6 – 11.9 Y | 1. Clinical assessment 2. Growth measured | 0 | 0 | **Cushingoid features** | **23** | **-** |
|  |  |  |  |  |  |  |  |  |  |  | **Skin striae** | **2** | **-** |
|  |  |  |  |  |  |  |  |  |  |  | **Infection** | **1** | **-** |
|  | Shiff et al. 2013 (81) | Canada | Rheumatic Diseases | Prednisolone | 2 – 52 W | 130 | 5.9 – 13.8 Y | 1. Clinical assessment 2. Blood pressure measured 3. Glucose measured 4. Weighted measured 5. Growth measured 6. Ophthalmologist | 0 | 0 | **Weight gain** | **65** | **-** |
|  |  |  |  |  |  |  |  |  |  |  | **Growth retardation** | **56** | **-** |
| **CS** | Kivity et al. 2004 (82) | Israel | Infantile Spasms | Prednisolone | 6 M | 37 | 3 – 7 M | - | 0 | 0 | **Cushingoid features** | **37** | **-** |
|  |  |  |  |  |  |  |  |  |  |  | **Hypertension** | **19** | **-** |
|  |  |  |  |  |  |  |  |  |  |  | **Hypokalemia** | **19** | **-** |
|  |  |  |  |  |  |  |  |  |  |  | **Infection** | **10** | **-** |
|  | Kossoff et al. 2009 (83) | USA | Infantile Spasms | Prednisolone | 1 M | 15 | 3 – 19 M | - | 5 (Lack of response) | 0 | **Behaviour change** | **5** | **-** |
|  |  |  |  |  |  |  |  |  |  |  | **Weight gain** | **2** | **-** |
|  |  |  |  |  |  |  |  |  |  |  | **Hypertension** | **1** | **-** |
|  |  |  |  |  |  |  |  |  |  |  | **Oedema** | **1** | **-** |
|  | Mytinger et al. 2010 (84) | USA | Infantile Spasms | Prednisolone | 2 M | 10 | 3 – 7 M | - | 0 | 0 | **Hypertension** | **1** | **-** |
|  |  |  |  |  |  |  |  |  |  |  | **HPA axis suppression** | **1** | **-** |
|  | Job et al. 1985 (85) | France | Congenital adrenal hyperplasia | Dexamethasone | 6 – 74 M | 18 | 10 –18 M | - | 0 | 0 | **Skin striae** | **5** | **-** |
|  |  |  |  |  |  |  |  |  |  |  | **Cushingoid features** | **4** | **-** |
|  | Bakchine et al. 1984 (86) | France | Renal transplantation | Prednisolone | 3 – 6 M | 6 | 8 – 15 Y | - | 0 | 0 | **Hyperglycaemia** | **6** | **-** |
|  | Thacker et al. 2010 (87) | USA | Protein-Losing Enteropathy | Budesonide | 6 M | 9 | 1.2 – 10.8 Y | - | 0 | 0 | **Infection** | **5** | **-** |
|  |  |  |  |  |  |  |  |  |  |  | **Cushingoid features** | **3** | **-** |
|  |  |  |  |  |  |  |  |  |  |  | **Osteoporosis** | **3** | **-** |
|  |  |  |  |  |  |  |  |  |  |  | **Acne** | **1** | **-** |

**Abbreviation:** PCS, Prospective cohort studies. CS, Case series.

**Table 8 Summary of ADRs form Mixed Conditions (continued)**

| **Types of studies** | **Authors**  **and years** | **Country** | **Medical diagnosis** | **Drug name** | **Duration** | **No. of patients** | **Age** | **ADRs detect method** | **No. of pt. withdraw** | **Mortality** | **Side effects** | **No. of SE** | |
| --- | --- | --- | --- | --- | --- | --- | --- | --- | --- | --- | --- | --- | --- |
|  |  |  |  |  |  |  |  |  |  |  |  | **A** | **B** |
| **CS** | Schumacher et al. 2011 (88) | UAS | Protein-Losing Enteropathy | Budesonide | 6 M | 10 | 1.6 – 10 Y | - | 3 (Lack of response) | 0 | **Infection** | **5** | **-** |
|  |  |  |  |  |  |  |  |  |  |  | **Cushingoid features** | **3** | **-** |
|  |  |  |  |  |  |  |  |  |  |  | **Gastric Ulcer** | **1** | **-** |
|  |  |  |  |  |  |  |  |  |  |  | **Hirsutism** | **1** | **-** |
|  | Gursu et al. 2014 (89) | Turkey | Protein-Losing Enteropathy | Budesonide | 6 – 9 M | 4 | 3.5 – 6 Y | - | 0 | 0 | **Cushingoid features** | **3** | **-** |
|  |  |  |  |  |  |  |  |  |  |  | **Infection** | **1** | **-** |
| **CR** | Curless wt al. 1986 (90) | USA | Kearns-Sayre syndrome | Prednisolone | 2 – 3 W | 2 | 11 + 13 Y | - | 0 | 2 (Died) | **Hyperglycaemia** | **2** | **-** |
|  | Rejou et al. 1986 (91) | France | Dermatomyositis | Prednisolone | 9 – 13 M | 2 | 7 + 11 Y | - | 0 | 0 | **Osteoporosis** | **2** | **-** |
|  |  |  |  |  |  |  |  |  |  |  | **Growth retardation** | **1** | **-** |
|  | Story-Lewis et al. 1987 (92) | USA | Systemic lupus erythematosus | Prednisolone | 6 W | 1 | 7.5 Y | - | 0 | 0 | **Cushingoid features** | **1** | **-** |
|  |  |  |  |  |  |  |  |  |  |  | **Ulceration** | **1** | **-** |
|  |  |  |  |  |  |  |  |  |  |  | **Fever** | **1** | **-** |
|  |  |  |  |  |  |  |  |  |  |  | **Rash** | **1** | **-** |
|  | Splain and Berman 1992 (93) | USA | Diamond–Blackfan anaemia | Prednisolone | 3 – 4 M | 2 | 9 + 13 Y | - | 0 | 0 | **Cushingoid features** | **2** | **-** |
|  |  |  |  |  |  |  |  |  |  |  | **Growth retardation** | **2** | **-** |
|  |  |  |  |  |  |  |  |  |  |  | **Osteoporosis** | **2** | **-** |
|  | Jackson and Song 1995 (94) | South Africa | Autoimmune chronic active hepatitis | Prednisolone | 6 M | 1 | 17 Y | - | 0 | 0 | **Cushingoid features** | **1** | **-** |
|  |  |  |  |  |  |  |  |  |  |  | **Gastric ulcer** | **1** | **-** |
|  |  |  |  |  |  |  |  |  |  |  | **Pancreatitis** | **1** | **-** |
|  | Alessandri et al. 2000 (95) | Canada | Diamond–Blackfan anaemia | Prednisolone | 4 Y | 1 | 4 M | - | 0 | 0 | **Cushingoid features** | **1** | **-** |
|  |  |  |  |  |  |  |  |  |  |  | **Growth retardation** | **1** | **-** |
|  |  |  |  |  |  |  |  |  |  |  | **Infection** | **1** | **-** |
|  |  |  |  |  |  |  |  |  |  |  | **Headache** | **1** | **-** |
|  | Gupta et al. 2004 (96) | India | Pemphigus erythematosus | Prednisolone | 3 Y | 1 | 7 Y | - | 0 | 0 | **Cushingoid features** | **1** | **-** |
|  |  |  |  |  |  |  |  |  |  |  | **Osteoporosis** | **1** | **-** |
|  | Teixeira Jr et al. 2005 (97) | Brazil | Sydenham's chorea | Prednisolone | 1 Y | 1 | 12.8 Y | - | 0 | 0 | **Weight gain** | **1** | **-** |
|  |  |  |  |  |  |  |  |  |  |  | **Cushingoid features** | **1** | **-** |
|  | Ishigaki et al. 2009 (98) | Japan | Ocular myasthenia gravis | Prednisolone | 3 Y | 1 | 13 M | -  - | 0  0 | 0  0 | **Cushingoid features** | **1** | **-** |
|  |  |  |  |  |  |  |  |  |  |  | **Weight gain** | **1** | **-** |
|  |  |  |  |  |  |  |  |  |  |  | **Hypertension** | **1** | **-** |
|  |  |  |  |  |  |  |  |  |  |  | **Pigmentation** | **1** | **-** |
|  |  |  |  |  |  |  |  |  |  |  | **Cataract** | **1** | **-** |
|  |  |  |  |  |  |  |  |  |  |  | **Hirsutism** | **1** | **-** |
|  | Srinivasan et al. 2011 (99) | India | Hypereosinophilic syndrome | Prednisolone | 1 M | 1 | 11 Y | - | 0 | 0 | **Weight gain** | **1** | **-** |
|  |  |  |  |  |  |  |  |  |  |  | **Gastric ulcer** | **1** | **-** |
|  | Kim et al. 2013 (100) | Korea | Eosinophilic cellulitis | Prednisolone | 11 W | 1 | 11 Y | - | 1 (IOP) | 0 | **Increased intraocular pressure (IOP)** | **1** | **-** |
|  | Gursu et al. 2014 (101) | Turkey | Protein-Losing Enteropathy | Budesonide | 6 M | 1 | 7 Y | - | 0 | 0 | **Infection** | **1** | **-** |
|  |  |  |  |  |  |  |  |  |  |  | **Cushingoid features** | **1** | **-** |

**Abbreviation:** CS, Case series. CR, Case report.

**References**

1. Bostrom BC, Sensel MR, Sather HN, Gaynon PS, La MK, Johnston K, et al. Dexamethasone versus prednisone and daily oral versus weekly intravenous mercaptopurine for patients with standard-risk acute lymphoblastic leukemia: a report from the Children’s Cancer Group. Blood. 2003 May 15;101(10):3809–17.

2. Mitchell CD, Richards SM, Kinsey SE, Lilleyman J, Vora A, Eden TOB. Benefit of dexamethasone compared with prednisolone for childhood acute lymphoblastic leukaemia: results of the UK Medical Research Council ALL97 randomized trial. Br J Haematol. 2005 Jun;129(6):734–45.

3. Einaudi S, Bertorello N, Masera N, Farinasso L, Barisone E, Rizzari C, et al. Adrenal axis function after high-dose steroid therapy for childhood acute lymphoblastic leukemia. Pediatr Blood Cancer. 2008 Mar;50(3):537–41.

4. Lightner ES, Johnson H, Corrigan JJ. Rapid adrenocortical recovery after short-term glucocorticoid therapy. Am J Dis Child. 1981 Sep;135(9):790–2.

5. Drigan R, Spirito A, Gelber RD. Behavioral effects of corticosteroids in children with acute lymphoblastic leukemia. Med Pediatr Oncol. 1992 Jan;20(1):13–21.

6. Felner EI, Thompson MT, Ratliff AF, White PC, Dickson BA. Time course of recovery of adrenal function in children treated for leukemia. J Pediatr. 2000 Jul;137(1):21–4.

7. Kuperman H, Damiani D, Chrousos GP, Dichtchekenian V, Manna TD, Filho VO, et al. Evaluation of the hypothalamic-pituitary-adrenal axis in children with leukemia before and after 6 weeks of high-dose glucocorticoid therapy. J Clin Endocrinol Metab. 2001 Jul;86(7):2993–6.

8. Petersen KB, Müller J, Rasmussen M, Schmiegelow K. Impaired adrenal function after glucocorticoid therapy in children with acute lymphoblastic leukemia. Med Pediatr Oncol. 2003 Aug;41(2):110–4.

9. Mahachoklertwattana P, Vilaiyuk S, Hongeng S, Okascharoen C. Suppression of adrenal function in children with acute lymphoblastic leukemia following induction therapy with corticosteroid and other cytotoxic agents. J Pediatr. 2004 Jun;144(6):736–40.

10. Cunha C de F, Silva IN, Finch FL. Early adrenocortical recovery after glucocorticoid therapy in children with leukemia. J Clin Endocrinol Metab. 2004 Jun;89(6):2797–802.

11. Rix M, Birkebaek NH, Rosthøj S, Clausen N. Clinical impact of corticosteroid-induced adrenal suppression during treatment for acute lymphoblastic leukemia in children: a prospective observational study using the low-dose adrenocorticotropin test. J Pediatr. 2005 Nov;147(5):645–50.

12. Gordijn MS, van Litsenburg RR, Gemke RJBJ, Bierings MB, Hoogerbrugge PM, van de Ven PM, et al. Hypothalamic-pituitary-adrenal axis function in survivors of childhood acute lymphoblastic leukemia and healthy controls. Psychoneuroendocrinology. 2012 Sep;37(9):1448–56.

13. Elliott AJ, Oakhill A, Goodman S. Cataracts in childhood leukaemia. Br J Ophthalmol. 1985 Jun;69(6):459–61.

14. Matsuzaki A, Suminoe A, Koga Y, Kusuhara K, Hara T, Ogata R, et al. Fatal visceral varicella-zoster virus infection without skin involvement in a child with acute lymphoblastic leukemia. Pediatr Hematol Oncol. 2008;25(3):237–42.

15. Watanabe TK, Sylvester CE, Manaligod JM. Mania or panic associated with dexamethasone chemotherapy in adolescents. J Adolesc Health. 1994 Jun;15(4):345–7.

16. Pope E, Krafchik BR, Macarthur C, Stempak D, Stephens D, Weinstein M, et al. Oral versus high-dose pulse corticosteroids for problematic infantile hemangiomas: a randomized, controlled trial. Pediatrics. 2007 Jun;119(6):e1239-47.

17. Bauman NM, McCarter RJ, Guzzetta PC, Shin JJ, Oh AK, Preciado DA, et al. Propranolol vs prednisolone for symptomatic proliferating infantile hemangiomas: a randomized clinical trial. JAMA Otolaryngol Head Neck Surg. 2014 Apr;140(4):323–30.

18. Aly MMD, Hamza AF, Abdel Kader HM, Saafan HA, Ghazy MS, Ragab IA. Therapeutic superiority of combined propranolol with short steroids course over propranolol monotherapy in infantile hemangioma. Eur J Pediatr. 2015 Nov;174(11):1503–9.

19. Boon LM, MacDonald DM, Mulliken JB. Complications of systemic corticosteroid therapy for problematic hemangioma. Plast Reconstr Surg. 1999 Nov;104(6):1616–23.

20. Pandey A, Gangopadhyay AN, Gopal SC, Kumar V, Sharma SP, Gupta DK, et al. Twenty years’ experience of steroids in infantile hemangioma--a developing country’s perspective. J Pediatr Surg. 2009 Apr;44(4):688–94.

21. Jalil A, Maino A, Bhojwani R, Vose M, Ashworth J, Lloyd IC, et al. Clinical review of periorbital capillary hemangioma of infancy. J Pediatr Ophthalmol Strabismus. 2011;48(4):218–25.

22. Bertrand J, McCuaig C, Dubois J, Hatami A, Ondrejchak S, Powell J. Propranolol versus prednisone in the treatment of infantile hemangiomas: a retrospective comparative study. Pediatr Dermatol. 2011;28(6):649–54.

23. Grover C, Kedar A, Arora P, Lal B. Efficacy of oral prednisolone use in the treatment of infantile hemangiomas in Indian children. Pediatr Dermatol. 2011;28(5):502–6.

24. Price CJ, Lattouf C, Baum B, McLeod M, Schachner LA, Duarte AM, et al. Propranolol vs corticosteroids for infantile hemangiomas: a multicenter retrospective analysis. Arch Dermatol. 2011 Dec;147(12):1371–6.

25. Enjolras O, Riche MC, Merland JJ, Escande JP. Management of alarming hemangiomas in infancy: a review of 25 cases. Pediatrics. 1990 Apr;85(4):491–8.

26. Sadan N, Wolach B. Treatment of hemangiomas of infants with high doses of prednisone. J Pediatr. 1996 Jan;128(1):141–6.

27. Uysal KM, Olgun N, Erbay A, Sarialioğlu F. High-dose oral methylprednisolone therapy in childhood hemangiomas. Pediatr Hematol Oncol. 2001;18(5):335–41.

28. Mallo S, Torrelo A, Zambrano A. Treatment of hemangiomas in children with oral corticosteroids. [Spanish] Tratamiento de hemangiomas infantiles con corticoides orales. Actas Dermosifiliogr. 2004;95(6):370–3.

29. Corapcioğlu F, Büyükkapu-Bay S, Binnetoğlu K, Babaoğlu A, Anik Y, Tugay M. Preliminary results of propranolol treatment for patients with infantile hemangioma. Turk J Pediatr. 2011;53(2):137–41.

30. Nieuwenhuis K, de Laat PCJ, Janmohamed SR, Madern GC, Oranje AP. Infantile hemangioma: treatment with short course systemic corticosteroid therapy as an alternative for propranolol. Pediatr Dermatol. 2013;30(1):64–70.

31. Herrero Hernández A, Escobosa Sánchez O, Acha García T. Successful treatment with vincristine in PHACES syndrome. Clin Transl Oncol. 2007 Apr;9(4):262–3.

32. Koay ACA, Choo MM, Nathan AM, Omar A, Lim CT. Combined low-dose oral propranolol and oral prednisolone as first-line treatment in periocular infantile hemangiomas. J Ocul Pharmacol Ther. 2011 Jun;27(3):309–11.

33. Morais P, Magina S, Mateus M, Trindade E, Jesus JM, Azevedo F. Efficacy and safety of propranolol in the treatment of parotid hemangioma. Cutan Ocul Toxicol. 2011 Sep;30(3):245–8.

34. Wolthers OD, Pedersen S. Short term linear growth in asthmatic children during treatment with prednisolone. BMJ. 1990 Jul 21;301(6744):145–8.

35. Rooklin AR, Lampert SI, Jaeger EA, McGeady SJ, Mansmann HC. Posterior subcapsular cataracts in steroid-requiring asthmatic children. J Allergy Clin Immunol. 1979 Jun;63(6):383–6.

36. Wiersbitzky S, Ballke EH, Patzschke V. The growth of children with bronchial asthma treated by different therapeutic regimens. [German] Das Somatische Gedeihen Von Kindern Mit Asthma Bronchiale Unter Verschiedenen Therapie-Regimen. Arztl Jugendkd. 1982;73(4):225–32.

37. Nassif E, Weinberger M, Sherman B, Brown K. Extrapulmonary effects of maintenance corticosteroid therapy with alternate-day prednisone and inhaled beclomethasone in children with chronic asthma. J Allergy Clin Immunol. 1987 Oct;80(4):518–29.

38. Kelly HW, Van Natta ML, Covar RA, Tonascia J, Green RP, Strunk RC. Effect of long-term corticosteroid use on bone mineral density in children: a prospective longitudinal assessment in the childhood Asthma Management Program (CAMP) study. Pediatrics. 2008 Jul;122(1):e53-61.

39. Hawcutt DB, Jorgensen AL, Wallin N, Thompson B, Peak M, Lacy D, et al. Adrenal responses to a low-dose short synacthen test in children with asthma. Clin Endocrinol (Oxf). 2015 May;82(5):648–56.

40. Sanders BP, Portman RJ, Ramey RA, Hill M, Strunk RC. Hypertension during reduction of long-term steroid therapy in young subjects with asthma. J Allergy Clin Immunol. 1992 Apr;89(4):816–21.

41. Silk HJ, Guay-Woodford L, Perez-Atayde AR, Geha RS, Broff MD. Fatal varicella in steroid-dependent asthma. J Allergy Clin Immunol. 1988 Jan;81(1):47–51.

42. Zychowicz C, Wicinska W. A successful attempt at replacing prednisone and kenalog with inhalations of beclomethasone dipropionate in 2 girls with bronchial asthma and drug-induced growth arrest. [Polish]. Wiad Lek. 1982;35(18):1177–80.

43. Abuekteish F, Kirkpatrick JN, Russell G. Posterior subcapsular cataract and inhaled corticosteroid therapy. Thorax. 1995 Jun;50(6):674–6.

44. Broyer M, Terzi F, Lehnert A, Gagnadoux MF, Guest G, Niaudet P. A controlled study of deflazacort in the treatment of idiopathic nephrotic syndrome. Pediatr Nephrol. 1997 Aug;11(4):418–22.

45. Miltenyi M, Homoki J, Fazekas AK. Posterior subcapsular cataracts, associated with long-term corticosteroid therapy. Prednisolone versus 6alpha-fluor-16alpha-methyl-1-dehydrocorticosterone. Helv Paediatr Acta. 1983;38(2):141–7.

46. Foote KD, Brocklebank JT, Meadow SR. Height attainment in children with steroid-responsive nephrotic syndrome. Lancet. 1985;2(8461):917–9.

47. Merritt RJ, Hack SL, Kalsch M, Olson D. Corticosteroid therapy-induced obesity in children. Clin Pediatr (Phila). 1986 Mar;25(3):149–52.

48. Garin EH, Sleasman JW, Richard GA, Iravani AA, Fennell RS. Pulsed methylprednisolone therapy compared to high dose prednisone in systemic lupus erythematosus nephritis. Eur J Pediatr. 1986 Oct;145(5):380–3.

49. Nammalwar BR, Vijaykumar M, Prahlad N, Jain D V. Steroid resistant nephrotic syndrome is sustained remission attainable. Indian Pediatr. 2006 Jan;43(1):39–43.

50. Bahat E, Akkaya BK, Akman S, Karpuzoglu G, Guven AG. Comparison of pulse and oral steroid in childhood membranoproliferative glomerulonephritis. J Nephrol. 2007;20(2):234–45.

51. Upadhyay A, Mishra OP, Prasad R, Upadhyay SK, Schaefer F. Behavioural abnormalities in children with new-onset nephrotic syndrome receiving corticosteroid therapy: results of a prospective longitudinal study. Pediatr Nephrol. 2016 Feb;31(2):233–8.

52. Rizzoni G, Todesco L, Callegari M. Long-term alternate-day steroid treatment of frequently relapsing nephrotic syndrome. [Italian] La Corticoterapia Discontinua Prolungata (Cdp) Nel Trattamento Della Sindrome Nefrosica a Frequenti Recidive. Riv Ital di Pediatr. 1986;12(5):519–22.

53. Fujita K, Sanae N, Yoshioka H, Kurokawa H, Sato T. Steroid Therapy and Fatal Varicella in a Nephrotic Patient; A Necropsy case with Atypical Rash. Pediatr Int. 1983;25(3):322–5.

54. George Jr WE, Wilmot M, Greenhouse A, Hammeke M. Medical management of steroid-induced epidural lipomatosis. N Engl J Med. 1983;308(6):316–9.

55. Kaneda A, Yamaura I, Kamikozuru M, Nakai O. Paraplegia as a complication of corticosteroid therapy. J Bone Jt Surg - Ser A. 1984;66(5):783–5.

56. Tsuruga K, Eishin OKI, Suzuki K, Takahashi Y, Tanaka H. Early initiation of peritoneal dialysis for the treatment of a patient with refractory nephrotic syndrome. [Japanese]. Japanese J Nephrol. 2009;51(1):68–73.

57. Greally P, Hussain MJ, Vergani D, Price JF. Interleukin-1 alpha, soluble interleukin-2 receptor, and IgG concentrations in cystic fibrosis treated with prednisolone. Arch Dis Child. 1994 Jul;71(1):35–9.

58. Eigen H, Rosenstein BJ, FitzSimmons S, Schidlow D V. A multicenter study of alternate-day prednisone therapy in patients with cystic fibrosis. Cystic Fibrosis Foundation Prednisone Trial Group. J Pediatr. 1995 Apr;126(4):515–23.

59. Ruuska T, Savilahti E, Mäki M, Ormälä T, Visakorpi JK. Exclusive whole protein enteral diet versus prednisolone in the treatment of acute Crohn’s disease in children. J Pediatr Gastroenterol Nutr. 1994 Aug;19(2):175–80.

60. Levine A, Weizman Z, Broide E, Shamir R, Shaoul R, Pacht A, et al. A comparison of budesonide and prednisone for the treatment of active pediatric Crohn disease. J Pediatr Gastroenterol Nutr. 2003 Feb;36(2):248–52.

61. Escher JC. Budesonide versus prednisolone for the treatment of active Crohn’s disease in children: a randomized, double-blind, controlled, multicentre trial. Eur J Gastroenterol Hepatol. 2004 Jan;16(1):47–54.

62. Levine A, Kori M, Dinari G, Broide E, Shaoul R, Yerushalmi B, et al. Comparison of two dosing methods for induction of response and remission with oral budesonide in active pediatric Crohn’s disease: a randomized placebo-controlled trial. Inflamm Bowel Dis. 2009 Jul;15(7):1055–61.

63. Wales JK, Milner RD. Variation in lower leg growth with alternate day steroid treatment. Arch Dis Child. 1988 Aug;63(8):981–3.

64. Imbach P, Wagner HP, Berchtold W, Gaedicke G, Hirt A, Joller P, et al. Intravenous immunoglobulin versus oral corticosteroids in acute immune thrombocytopenic purpura in childhood. Lancet. 1985 Aug 31;2(8453):464–8.

65. Blanchette V, Imbach P, Andrew M, Adams M, McMillan J, Wang E, et al. Randomised trial of intravenous immunoglobulin G, intravenous anti-D, and oral prednisone in childhood acute immune thrombocytopenic purpura. Lancet. 1994 Sep 10;344(8924):703–7.

66. Chen JS, Wu JM, Chen YJ, Yeh TF. Pulsed high-dose dexamethasone therapy in children with chronic idiopathic thrombocytopenic purpura. J Pediatr Hematol Oncol. 1997;19(6):526–9.

67. Kühne T, Freedman J, Semple JW, Doyle J, Butchart S, Blanchette VS. Platelet and immune responses to oral cyclic dexamethasone therapy in childhood chronic immune thrombocytopenic purpura. J Pediatr. 1997 Jan;130(1):17–24.

68. Borgna-Pignatti C, Rugolotto S, Nobili B, Amendola G, De Stefano P, Maccario R, et al. A trial of high-dose dexamethasone therapy for chronic idiopathic thrombocytopenic purpura in childhood. J Pediatr. 1997 Jan;130(1):13–6.

69. Wali YA, Al Lamki Z, Shah W, Zacharia M, Hassan A. Pulsed high-dose dexamethasone therapy in children with chronic idiopathic thrombocytopenic purpura. Pediatr Hematol Oncol. 2002;19(5):329–35.

70. Sakurai Y, Ohkubo Y, Miura S, Mariko M, Akazawa H, Imanaka Y, et al. Liposteroid therapy for chronic childhood idiopathic thrombocytopenic purpura: Two case reports. Int J Pediatr Hematol. 1999;6(1):27–31.

71. Fenichel GM, Mendell JR, Moxley RT, Griggs RC, Brooke MH, Miller JP, et al. A comparison of daily and alternate-day prednisone therapy in the treatment of Duchenne muscular dystrophy. Arch Neurol. 1991 Jun;48(6):575–9.

72. Zulian F, Martini G, Vallongo C, Vittadello F, Falcini F, Patrizi A, et al. Methotrexate treatment in juvenile localized scleroderma: a randomized, double-blind, placebo-controlled trial. Arthritis Rheum. 2011 Jul;63(7):1998–2006.

73. Ozturk F, Bakirtas A, Ileri F, Turktas I. Efficacy and tolerability of systemic methylprednisolone in children and adolescents with chronic rhinosinusitis: a double-blind, placebo-controlled randomized trial. J Allergy Clin Immunol. 2011 Aug;128(2):348–52.

74. Zannolli R, Buoni S, Betti G, Salvucci S, Plebani A, Soresina A, et al. A randomized trial of oral betamethasone to reduce ataxia symptoms in ataxia telangiectasia. Mov Disord. 2012 Sep 1;27(10):1312–6.

75. Woynarowski M, Nemeth A, Baruch Y, Koletzko S, Melter M, Rodeck B, et al. Budesonide versus prednisone with azathioprine for the treatment of autoimmune hepatitis in children and adolescents. J Pediatr. 2013 Nov;163(5):1347–53.e1.

76. Dowell SF, Bresee JS. Severe varicella associated with steroid use. Pediatrics. 1993 Aug;92(2):223–8.

77. Connolly AM, Schierbecker J, Renna R, Florence J. High dose weekly oral prednisone improves strength in boys with Duchenne muscular dystrophy. Neuromuscul Disord. 2002 Dec;12(10):917–25.

78. Ait Ourhroui M, Hassam B, Khoudri I. [Treatment of alopecia areata with prednisone in a once-monthly oral pulse]. Ann Dermatol Venereol. 2010;137(8–9):514–8.

79. Miura M, Tamame T, Naganuma T, Chinen S, Matsuoka M, Ohki H. Steroid pulse therapy for Kawasaki disease unresponsive to additional immunoglobulin therapy. Paediatr Child Health. 2011 Oct;16(8):479–84.

80. Torok KS, Arkachaisri T. Methotrexate and corticosteroids in the treatment of localized scleroderma: a standardized prospective longitudinal single-center study. J Rheumatol. 2012 Feb;39(2):286–94.

81. Shiff NJ, Brant R, Guzman J, Cabral DA, Huber AM, Miettunen P, et al. Glucocorticoid-related changes in body mass index among children and adolescents with rheumatic diseases. Arthritis Care Res (Hoboken). 2013 Jan;65(1):113–21.

82. Kivity S, Lerman P, Ariel R, Danziger Y, Mimouni M, Shinnar S. Long-term cognitive outcomes of a cohort of children with cryptogenic infantile spasms treated with high-dose adrenocorticotropic hormone. Epilepsia. 2004 Mar;45(3):255–62.

83. Kossoff EH, Hartman AL, Rubenstein JE, Vining EPG. High-dose oral prednisolone for infantile spasms: an effective and less expensive alternative to ACTH. Epilepsy Behav. 2009 Apr;14(4):674–6.

84. Mytinger JR, Quigg M, Taft WC, Buck ML, Rust RS. Outcomes in treatment of infantile spasms with pulse methylprednisolone. J Child Neurol. 2010 Aug;25(8):948–53.

85. Job JC, Munck A, Chaussain JL, Canlorbe P. Effects and side-effects of dexamethasone used as treatment of congenital virilizing adrenal hyperplasia in adolescent patients. [French] Traitement De L’hyperplasie Surrenale Virilisante Chez Les Adolescents. Emploi Et Inconvenients De La Dexamethasone. Arch Fr Pediatr. 1985;42(9):765–9.

86. Bakchine H, Niaudet P, Gagnadoux MF. Steroid-induced diabetes in 6 children after renal transplantation. [French] Diabete Induit Par Les Corticoides. Chez Six Enfants Apres Transplantation Renale. Arch Fr Pediatr. 1984;41(4):261–4.

87. Thacker D, Patel A, Dodds K, Goldberg DJ, Semeao E, Rychik J. Use of oral budesonide in the management of protein-losing enteropathy after the Fontan operation. Ann Thorac Surg. 2010 Mar;89(3):837–42.

88. Schumacher KR, Cools M, Goldstein BH, Ioffe-Dahan V, King K, Gaffney D, et al. Oral budesonide treatment for protein-losing enteropathy in Fontan-palliated patients. Pediatr Cardiol. 2011 Oct;32(7):966–71.

89. Gursu HA, Erdogan I, Varan B, Oktay A, Ozcay F, Ozkan M, et al. Oral budesonide as a therapy for protein-losing enteropathy in children after the Fontan operation. J Card Surg. 2014 Sep;29(5):712–6.

90. Curless RG, Flynn J, Bachynski B, Gregorios JB, Benke P, Cullen R. Fatal metabolic acidosis, hyperglycemia, and coma after steroid therapy for Kearns-Sayre syndrome. Neurology. 1986 Jun;36(6):872–3.

91. Rejou F, Dumas R, Belon C, Meunier PJ, Edouard C. Fluoride treatment in corticosteroid induced osteoporosis. Arch Dis Child. 1986 Dec;61(12):1230–1.

92. Van Story-Lewis PE, Roberts MW, Klippel JH. Oral effects of steroid therapy in a patient with systemic lupus erythematosus: report of case. J Am Dent Assoc. 1987;115(1):49–51.

93. Splain J, Berman BW. Cyclosporin A treatment for Diamond-Blackfan anemia. Am J Hematol. 1992;39(3):208–11.

94. Jackson LD, Song E. Cyclosporin in the treatment of corticosteroid resistant autoimmune chronic active hepatitis. Gut. 1995;36(3):459–61.

95. Alessandri AJ, Rogers PC, Wadsworth LD, Davis JH. Diamond-blackfan anemia and cyclosporine therapy revisited. J Pediatr Hematol Oncol. 2000;22(2):176–9.

96. Gupta MT, Jerajani HR. Control of childhood pemphigus erythematosus with steroids and azathioprine. Br J Dermatol. 2004 Jan;150(1):163–4.

97. Teixeira Jr AL, Maia DP, Cardoso F. Treatment of acute Sydenham’s chorea with methyl-prednisolone pulse-therapy. Parkinsonism Relat Disord. 2005 Aug;11(5):327–30.

98. Ishigaki K, Shishikura K, Murakami T, Suzuki H, Hirayama Y, Osawa M. Benefits of FK 506 for refractory eye symptoms in a young child with ocular myasthenia gravis. Brain Dev. 2009 Sep;31(8):634–7.

99. Srinivasan A, Lavanya R, Sankar J. Steroid-unresponsive hypereosinophilic syndrome. Ann Trop Paediatr. 2011 Jan;31(3):273–7.

100. Kim SH, Kwon JE, Kim H-B. Successful treatment of steroid-dependent eosinophilic cellulitis with cyclosporine. Allergy Asthma Immunol Res. 2013 Jan;5(1):62–4.

101. Gursu HA, Varan B, Erdogan I. Use of oral budesonide in the management of protein-losing enteropathy due to restrictive cardiomyopathy. Cardiol Young. 2014 Aug;24(4):764–6.
